# Measuring technical variability in illumina DNA methylation microarrays

**DOI:** 10.1371/journal.pone.0326337

**Published:** 2025-07-11

**Authors:** Anderson A. Butler, Jason J. Kras, Karolina P. Chwalek, Enrique I. Ramos, Isaac J. Bishof, David S. Vogel, Daniel L. Vera

**Affiliations:** 1 VoLo Foundation, Jupiter, Florida,; 2 Voloridge Health, Jupiter, Florida; Kore University of Enna: Universita degli Studi di Enna 'Kore', ITALY

## Abstract

DNA methylation microarrays have become a widely used tool for investigating epigenetic modifications in various aspects of biomedical research. However, technical variability in methylation data poses challenges for downstream applications such as predictive modeling of health and disease. In this study, we measure the impact of common sources of technical variability in Illumina DNA methylation microarray data, with a specific focus on positional biases inherent within the microarray technology. By utilizing a dataset comprised of multiple, highly similar technical replicates, we identified a chamber number bias, with different chambers of the microarray exhibiting systematic differences in fluorescence intensities (FI) and their derived methylation beta values, which are only partially corrected for by existing preprocessing methods and demonstrate that this positional bias can lead to false positive results during differential methylation testing. Additionally, our investigation identified outliers in low-level fluorescence data which might play a role in contributing to predictive error in computational models of health-relevant traits such as age.

## Introduction

In recent years, DNA methylation (DNAm) has received considerable interest as a potential biomarker for numerous clinically relevant traits, including disease and health risks [1–7]. In oncology, DNAm microarray-based assays which predict tumor site of origin have begun to see approved clinical use in the US and European Union for diagnostic purposes and for classifying cancers of unknown primary origin [8–10], and numerous methylation-derived risk scores (MRS) and predictive models for traits such as smoking status, body weight, and age are well-established in the scientific literature [8].

Improvements to analytical pipelines for array-based measurement of DNAm remain a high priority within the field, as technical limitations to data quality and consistency across batches currently interfere with the reliable detection of differences in DNAm, limiting the feasibility of DNAm microarray technologies for both research and clinical applications [8,11]. There is a general agreement regarding the need to account for batch and positional effects and other technical artifacts which influence microarray data and might otherwise result in spurious relationships [8]. To this end, many preprocessing methods have been developed which reduce the impact of positional effects and improve reliability. Several systematic studies have been undertaken to describe the efficacy and suitability of these methods in varying use cases. However, these studies are reliant on *in silico* experiments conducted either on repurposed experimental data or on simulated datasets. While highly valuable, conclusions drawn from such datasets must be interpreted with caution, given the potential for confounding latent variables in experimental data and potential discrepancies between simulated and real-world data.

Previous studies have examined the scale of technical noise within microarray experiments, primarily by measuring the intra-class correlation (ICC) as a metric of reliability for microarray probes. As the use of technical duplicate samples is optimal for studies aiming to measure ICC [12,13], these studies have limited applicability to those seeking to understand technical variability within microarrays [14].

While useful and informative, the wider utilization of established ICCs is limited by the metric itself, which measures technical variability only as it relates to biological variability for each CpG. However, as several authors examining the metric have noted, CpGs with presumably small levels of technical variability are often reported as unreliable due to the very low levels of biological variability for many CpGs within a single study and tissue type [14–17]. As ICC for any given CpG might be expected to increase, perhaps dramatically, in studies examining multiple tissue types, populations, or importantly, disease states [18], alternative metrics for measuring variability and reproducibility are beginning to be explored within the field [19]. Here, we explore the technical variability of Illumina microarray technology with an experimental approach that allows for the measurement of technical variability independently of biological variability.

Here, we characterized technical variability in Illumina’s MethylationEPICv1 microarray with an experimental design which allows us to isolate and measure the effect of multiple sources of technical variability. We directly examine the contribution of common positional effects to technical variability in methylation beta values within a set of highly similar replicates using linear mixed effects models and assess the performance of preprocessing in correcting for measurement differences across technical replicates. We explore the implications of our findings regarding technical variability and alternative preprocessing methods in the context of their application to experimental design, by examining the introduction and elimination of false positives as well as the impact of preprocessing on detectable biological variability with the data. Further, we examine the impact of preprocessing on well-accepted modeling targets in the literature: epigenetic age clocks.

## Results

### Experimental design to measure technical variability in Illumina’s MethylationEPIC microarrays

We first sought to design an experiment that would allow a comparison of positional effects in Illumina’s DNA methylation microarrays. We concentrated on isolating the chamber numbers (commonly known as Sentrix Positions) and slides (Sentrix Barcodes) to measure the contribution of positional effects to the variability observed among technical replicates.

**Fig 1:**
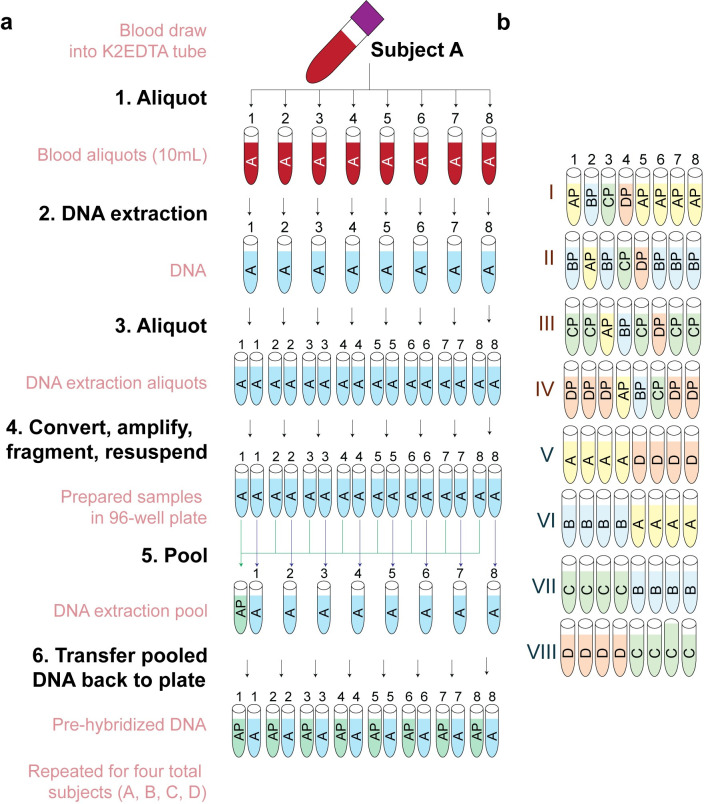
Schematic of experimental design to measure stability of microarray experiments. (A) Schematic of experimental design. Briefly, for each human subject, DNA was isolated from eight replicate blood aliquots, and then independently bisulfite converted, amplified, and frag-mented. An aliquot of DNA from each of the eight replicates was combined into a pooled DNA sample. Both independently isolated and pooled tech-nical replicates were measured across using Infinium MethylationEPIC microarrays. (B) Layout of pooled and independently prepared samples from each subject (Letters) with regard to chamber number/Sentrix posi-tion (numbers) within each slide/Sentrix Barcode (Roman numerals). Samples were used for independent statistical assessments as indicated.

For each of four human subjects, a single specimen of venous whole blood was collected and dispersed into aliquots prior to short-term frozen storage. Subsequently, eight of the aliquots for each subject were processed to isolate DNA for hybridization to microarrays, and then further split into two aliquots prior to deamination, amplification, and fragmentation (Fig 1). A total of eight samples, one from each of the DNA extractions, were then combined to form a pooled sample for each subject. Finally, eight aliquots from each subject’s pool and eight independently isolated technical replicates for each subject were profiled using Illumina EPIC v1 microarrays, for a total of 16 assays per subject.

To ensure that all positional effects were assayed, we measured each pooled replicate once in every chamber number, and at least once on each of four slides. To further examine the relative impact of sample preparation on variability, we also assayed eight independently prepared (unpooled) technical replicates for each subject, bring the total number of technical replicates per subject to 16 (Fig 1). These additional samples were organized for the purposes of examining the impact of positional effects on differential methylation testing.

### Multivariate analysis and statistical comparison of positional effects on CpGs in the illumina MethylationEPIC platform

**Fig 2:**
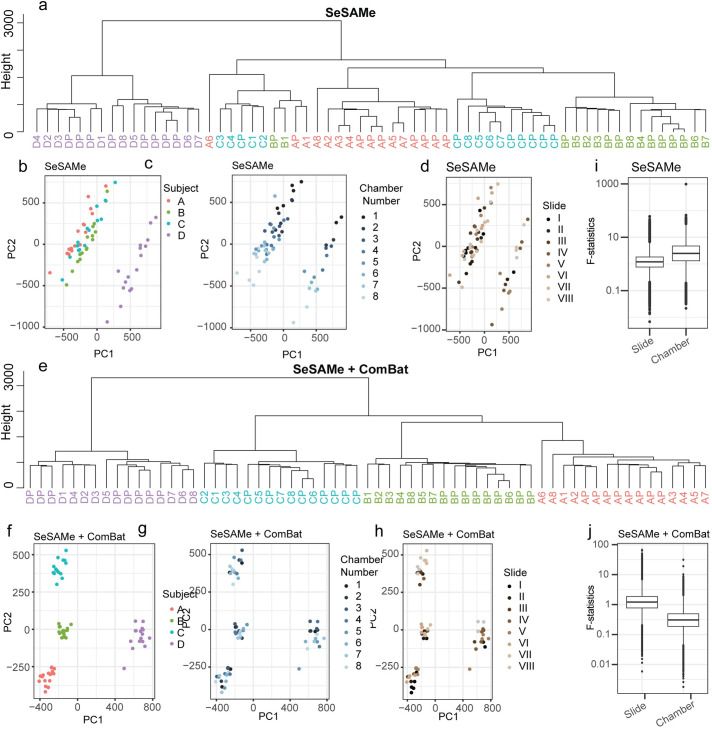
Multivariate Analysis and Statistical Comparison of Positional Effects on CpGs in the Illumina MethylationEPIC platform (A) Hierarchical clustering dendrogram representing the clustering of subjects based on the first 50 principal components from methylation beta values after preprocessing with SeSAMe. The vertical axis represents the dissimilarity between subjects, while the horizontal axis shows the subjects’ labels. (B) Principal component analysis (PCA) plot, representing the first principal component (PC1) against the second principal component (PC2) for each sample. Each data point represents an individual technical replicate, with colors indicating different subjects. (C-D) PCA plots showing the first two principal com-ponents for each sample, with colors indicating different chamber numbers (C) or different slides (D). (E) Hierarchical clustering dendrogram represent-ing the clustering of subjects based on the first 50 principal components from adjusted beta values after preprocessing with SeSAMe and ComBat adjust-ments for chamber number. (F-H) PCA plots showing clustering of subjects after ComBat adjustment for chamber number, colored by subject (F), cham-ber number (G), or array (H). (I-J) Box plots presenting F statistics obtained from analysis of variance (ANOVA) performed on beta values preprocessed either with SeSAMe alone (I) or with SeSAMe + ComBat using chamber number as batch (J). The box represents the interquartile range (IQR), with the median indicated by a line inside the box. Whiskers extend to the minimum and maximum values within 1.5 times the IQR. Data points beyond 1.5 times the IQR are plotted individually.

To begin to identify the main sources of variation within the Illumina MethylationEPIC dataset, we first conducted a principal component analysis (PCA) on beta values generated with the widely used preprocessing tool ‘SeSAMe’, using the authors’ recommended settings. Using the first fifty principal components as input, we performed hierarchical clustering of technical replicates. As each of the 16 total samples for each human subject were derived from a single blood draw (8 pooled plus 8 independently processed technical replicates), with the degrees of difference between them solely dependent upon technical factors, we anticipated that replicates from the same subject would cluster together, with biological variation between them as the main source of variation. Surprisingly, while several clusters appeared to be segregated by subject, one cluster contained intermingled samples from multiple subjects, suggesting that technical noise in methylation data may obfuscate biological signal (Fig 2). This lack of separation by subject is notable within the first two principal components (Fig 2). Importantly, we noted stratification by chamber number, but not slide number, within the first two principal components (Fig 2).

Given the observed stratification, we hypothesized that some bias in the data had been introduced by positional effects, particularly chamber number. To explore this hypothesis, we used ComBat, a commonly used empirical Bayes method for removing technical variation in DNA methylation microarray data, to adjust DNA methylation beta values for either chamber number or slide number. After controlling for slide, we noted no improvement in segregation in hierarchical clustering (S1 Fig) or within the first two principal components (S1 Fig); rather, clustering appeared worsened, with subject D less cleanly segregated than in unadjusted data. In contrast, after controlling for chamber number, we noted a perfect segregation by subject identity, as well as improved biological signal within the first two principal components (Fig 2).

To gain a greater understanding of how each of the positional effects impacts variability within the Illumina MethylationEPIC microarray, we examined the contribution of these effects to variance in methylation beta values using linear mixed effects models.

For each CpG, we used linear mixed effects regression to estimate the proportion of variance in beta values explained by the chamber and slide number positional effects. To compare aggregate data from all CpGs, we recorded the F-statistic as a metric of the impact of these positional effects. We observed that in data preprocessed with SeSaMe, the explainable variance due to chamber number is larger than the effect of slide (Fig 2). Consistent with this observation, correcting for the chamber number with ComBat appears to result in a greater overall reduction in explainable noise than correcting for the effect of slide, at least in the present dataset (Figs 2 and S1).

### Differential methylation testing on alternatively preprocessed methylation beta values from technical replicates

Having examined the contributions of positional effects to principal components of beta values, we next sought to determine the degree to which preprocessing pipelines might reduce beta value variability from a practical standpoint.

To accomplish this, for each CpG, we examined the ratio of the standard deviation (SD) between same-subject technical replicates to the SD for all replicates within the study. While replicates originating from the same initial sample are expected to have minimal biological variability, blood samples are also expected to have a high degree of similarity across subjects. Thus, to account for CpGs with very low biological variability, we added a small offset (0.0001) to the denominator when calculating SD ratios.

**Fig 3:**
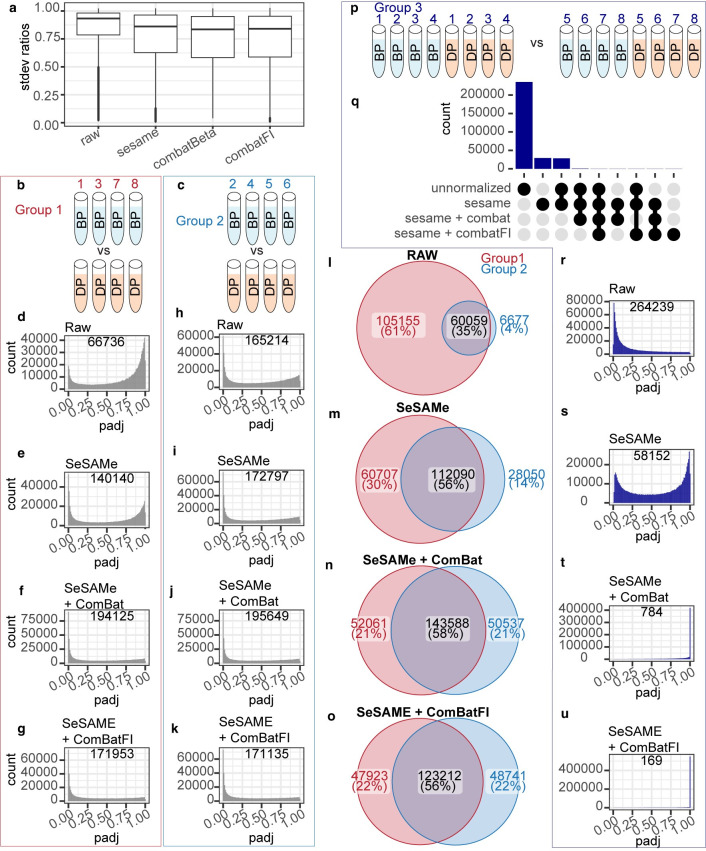
Differential methylation analysis of alternatively preprocessed methylation beta values from technical replicates. (A) Ratio of averaged standard deviation within subjects to standard deviation across all subjects for 614,831 CpGs shared across all preprocessing pipelines shown. (B-C) Representation of samples used for differential methylation testing in comparison group 1. Experimental subjects B and D were each represented by n = 4 technical replicates, with each replicate located on a different array for comparison group 1 (B) and each replicate on the same array for group 2 (C). (D-K) Histograms depicting BH adjusted p-values for all CpGs after differential methylation testing of beta values using the specified preprocessing pipeline for group 1 (in red box) and group 2 samples (in light blue box). Number of CpGs with BH-adjusted p-values < 0.05 listed on upper right of each plot. (L-O) Venn diagrams representing the number of CpGs detected as significantly different between subjects B and D in Group 1 (red circle), Group 2 (blue circle), or in both sets of samples (overlap), using the specified preprocessing pipeline. (P) Representation of samples used for differential methylation testing to screen for the introduction of false positives due to positional effects. (Q) Upset plot relating the number of false positives within and between preprocessing pipelines (limited to 9-most abundant comparisons). (R-U) Histograms depicting BH adjusted p-values for all CpGs after differential methylation testing of beta values using the specified preprocessing pipeline. Number of CpGs with BH-adjusted p-values < 0.05 listed on upper right of each plot.

We observed a marked reduction in SD ratio after using the authors’ recommended settings for SeSAMe, and a further reduction after correcting for the chamber number positional effect with either ComBat on beta values, or by correcting SeSAMe-adjusted FI using the ComBat-Seq algorithm (Fig 3).

There are reports in the literature that preprocessing pipelines, and in particular correcting for positional effects using the ComBat algorithm, might obfuscate biological variability or introduce false positives [20]. Therefore, we next sought to examine whether this was the case in our dataset. To accomplish this, we used the dmpFinder function from the minfi R package to conduct a series of differential methylation tests on subsets of samples (Figs 3 and S2).

As our data do not contain any positive controls, rather than test for false negatives, we instead examined the consistency between two independent differential methylation tests on groups of biologically different technical replicates (testing groups 1 & 2) as shown (Fig 3). As testing groups 1 & 2 are comprised of pooled technical replicates from the same two subjects, we are assured that any differences between the results of the two tests arise from technical noise rather than biological differences.

If preprocessing pipelines were masking biological signal, we would expect that the total number of differentially methylated CpGs observed would decrease relative to the number observed in the raw data. However, instead we observed that in both testing groups 1 & 2 the number of differentially methylated CpGs increases after preprocessing with SeSAMe, and is further increased by adjusting the beta values for positional effects with ComBat (Fig 3). Similarly, we find that the agreement between results is markedly improved with preprocessing using SeSAMe, and further improved by adjusting the beta values for positional effects with ComBat (Fig 3).

Next, we sought to examine whether preprocessing might introduce false positives as previously reported [20]. To test this hypothesis, we examined a third differential methylation test, in which technical replicates from two biological samples were equally represented across the groups being compared. As the underlying biological makeup across the comparison is identical, we assume that any statistically significant results from the analysis must be falsely positive.

We observed that many sites appear to be erroneously identified as differentially methylated when examining tests on raw or SeSAMe-adjusted beta values (Fig 3). While the number of false positives is improved after preprocessing using SeSaMe, many false positive results remain until correction for chamber number via ComBat (Fig 3). Interestingly, the greatest reduction in false positives in this assessment was achieved using the ComBat-seq algorithm [21], which we adapted to correct for positional effect on SeSAMe-adjusted FI (Fig 3).

Together, these results suggest that from a practical standpoint, the preprocessing pipelines tested here appear to reduce beta value variability and reduce the number of false positive results, while also preserving, or potentially unmasking, biological variability.

### Fluorescence and beta values are biased by positional effects in technical replicates

**Fig 4:**
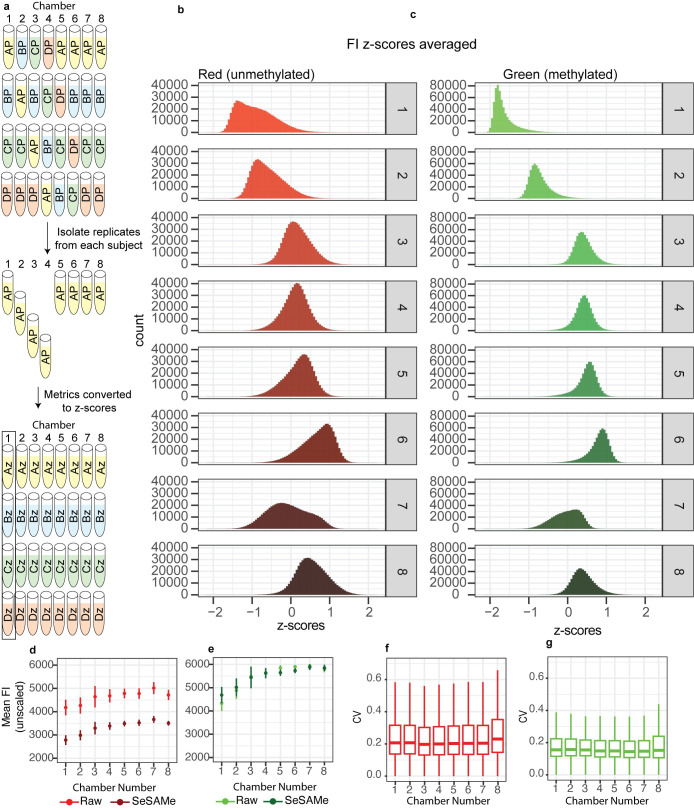
Fluorescence values compared in technical replicates measured in different chamber numbers. (A) Configuration of samples within slides/chambers. The average of four within-subject centered and scaled fluorescence intensity values for each CpG/chamber number were extracted from slides, as depicted. (B-C) Distribution histograms illustrating within-subject centered and scaled fluorescence intensities (FI) for all Type II probes on the MethylationEPIC array in the red (B) and green (C) channels. (D-E) Mean and standard deviation of fluorescence intensities (not centered or scaled) from all type 2 probes within in each chamber number. (F-G) Boxplots comparing the coefficients of variation from bead-level measurements (standard devia-tion/mean) for unprocessed FIs. Data represent all type 2 probes across each of the eight chamber numbers in the Red (F) and Green channels (G). Values for each chamber were calculated from measurements of four subjects, apart from chamber three which had one outlier sample removed based on quality concerns. Outlier values (>1.96 IQR) were hidden from boxplots due to the large size of the dataset.

Having established that chamber number corresponds to a large technical bias in beta values, we next sought to understand the origins of this bias. We first assessed low-level fluorescence data from each sample for differences in intensity. As the mean and variance for both FIs and beta values might be expected to differ between subjects for the same CpGs, we first centered and scaled values within technical replicates from the same subject, then averaged these centered and scaled values across four observations from different subjects as shown (Fig 4). To allow for a direct comparison between color channels, we limited our analysis of raw fluorescent intensities to Type II probes, in which the methylated and unmethylated signals are recorded on the same bead in the green and red channels, respectively. We noted differences in relative signal between chamber numbers in both the green and red fluorescent channels, with Fis recorded in chambers 1 and 2 appearing less bright than other chambers. Additionally, there were apparent differences in the distributions of values between the color channels, with chamber 1 appear right-skewed while some others appeared to have varying degrees of heteroskedasticity (Fig 4).

While differences between chambers might appear large when scaled, it might be possible that chamber number biases are magnified by the scaling process, as many probes have very low variability. To ascertain whether this might be the case, we averaged FIs of all probes in the red/green channels for each sample, and then averaged these sample means across all samples sharing the same chamber number, without centering or scaling. Similar to the results described above, we observed that the averaged mean FI in chamber number 1 was lower than in the other chambers, in both the red and green channels. Importantly, this bias in fluorescence intensities remained in both the green and red channel after preprocessing the data with SeSAMe (Figs 4 and S3 Fig).

As we observed differences in FI between chamber numbers, we suspected that the precision of measurements might also differ. To address this point, we calculated the coefficient of variation (CV) for each probe using the low-level mean FI and SD data from the raw idat files, and compared these across pooled samples. We noted that chamber 8 in particular displayed higher median CV than other chambers in the red channel (Fig 4). To deteremine whether this effect was restricted to some slides or present in all slides, we plotted each sample indepenedently and observed that this effect is limited to only two of the four slides examined in our dataset (S3 Fig). As this metric does not rely on the use of technical replicates, we sought to whether this effect was limited to our study, or if it was also present in external datasets. We examined the CV of FI measurements in five external datasets totaling over 1300 individual samples (S3 Fig). We noted heterogeneity of CV values between chamber numbers in all studies, and most commonly in chamber 8.

**Fig 5:**
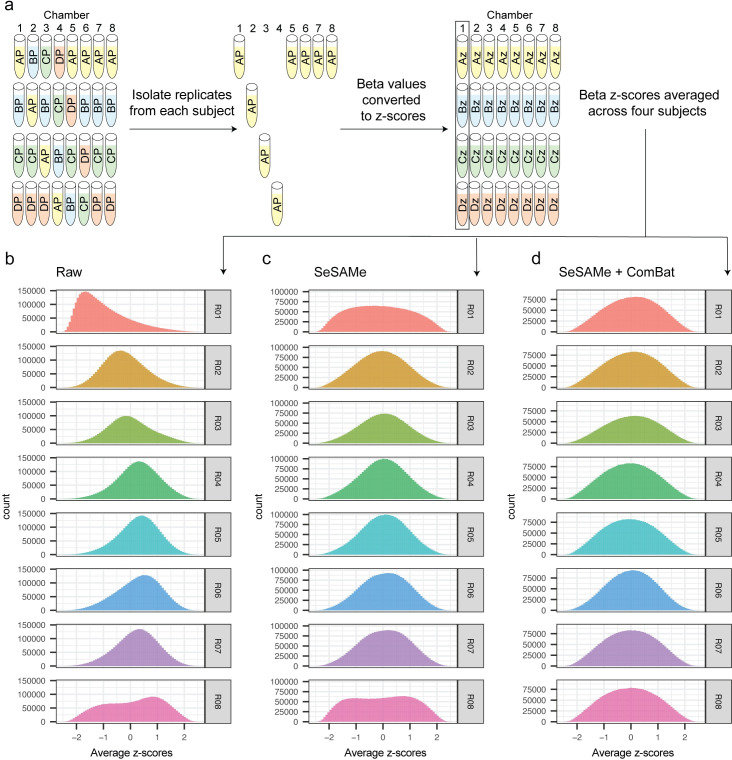
Beta values compared in technical replicates measured in different chamber numbers. (A) Configuration of samples within slides/chambers. The average of four within-subject centered and scaled beta values for each CpG/chamber number were extracted from slides, as de-picted. (B-D) Histograms representing the distributions of within-subject centered and scaled beta values for all probes on the MethylationEPIC array. The histograms correspond to different preprocessing steps: (B) raw beta values, (C) beta values preprocessed using SeSaMe’s recommended settings, and (D) SeSaMe-preprocessed beta values with adjustment for chamber number batch effects using ComBat.

We next sought to examine whether chamber number biases impacted beta values in our dataset, and indeed upon examining the averaged centered and scaled beta values from technical replicates, we observed a similar pattern as above, with technical replicates in chamber 1 and 2 appearing to show lower methylation overall than those in other chambers (Fig 5).

Notably, while preprocessing with SeSaMe’s recommended pipeline for the EPIC array appeared to center the distributions of beta values, the distribution of beta values for chambers 1 and 8 remained grossly different from the other chambers (Fig 5). Correcting SeSaMe-preprocessed data for chamber number batch effect with ComBat appeared to align the distributions for chambers 1 and 8 such that data from these chambers more closely resembled both data from other chambers as well as a normal distribution (Fig 5). To evaluate whether similar biases are present between different slides, we similarly converted beta values to within-subject z-scores and averaged scores across samples on the same slide, rather than chamber number (S4 Fig). While biases appear in raw beta values, we noted no gross differences in the distributions after preprocessing with SeSAMe (S4 Fig); notably, further adjusting for chamber number biases with ComBat appeared to have no detrimental effect on beta value distributions (S4 Fig).

### Interactions between fluorescence intensity/beta values and chamber number

Illumina Type 2 probes use the ratio of FI in the green (methylated) and red (unmethylated) channels to quantify the relative level of methylation for each genomic site. However, the intensity of fluorescence within each channel varies widely between probes. We next sought to examine whether probes with different FIs might be unequally impacted by positional effects. As the FI of samples from different subjects is expected to differ due to biological factors, we made within-subject comparisons to ascertain whether chamber number biases were larger in probes with a higher or lower FI.

**Fig 6:**
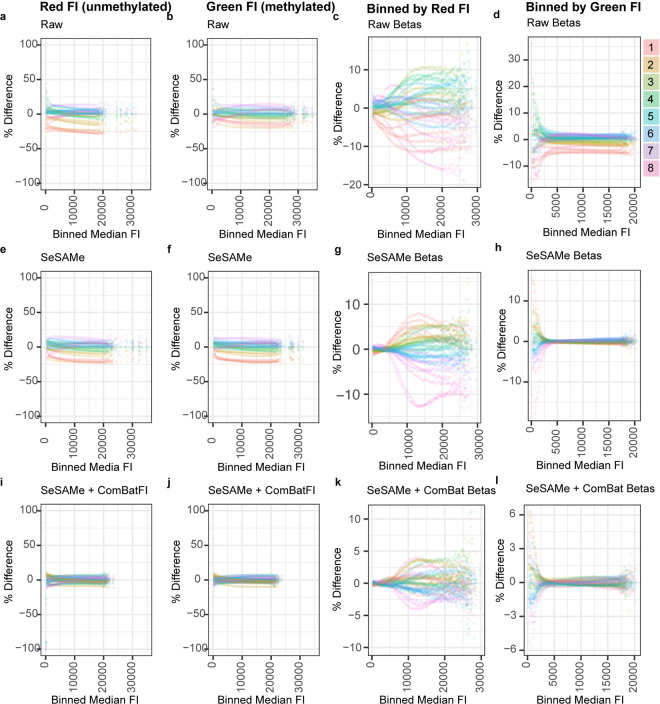
Relative brightness in technical replicates related to probe intensity. Plots depicting Type 2 probes binned according to median within-subject fluorescence intensity (FI). The y-axis represents differences from the median value for each bin. Each point shown represents one percentile bin from one of the four subjects in the study. Differences between median intensity are represented for the Red FI, Green FI, and beta values in Raw (A-D), SeSAMe-normalized (E-H), or ComBat-adjusted data (I-L). Axes for figure panels were intentionally limited for the purposes of visualization, resulting in the omission of some data points which had very high variability.

We first centered the FI measurements for each probe, by subtracting the median of all within-subject observations, and binned probes for each sample into quantiles by the median intensity within each color channel. Examining the FI within these bins shows the presence of an intensity-dependent bias, which increases in absolute magnitude in a manner directly proportional to the median FI in both the red and green channels (S5 Fig). We next asked whether the observed intensity-dependent chamber number bias increased proportionally with brightness in either the red or green color channels, or remained constant. In raw (Fig 6), SeSAMe-preprocessed (Fig 6), and SeSAMe + ComBat adjusted FIs (Fig 6), we observed consistent chamber number biases across all four subjects examined, which remained largely constant across the range of FIs in both the red and green channels.

As methylation beta values are ratiometrically derived from probe FIs, we next wondered whether positional biases might impact beta values in an intensity-dependent fashion. To answer this question, we first calculated the absolute deviation from the median FI for each probe, in each color channel. We then grouped probes into quantile bins by raw FI in either the green or red channel, and averaged the absolute deviation from median within each bin for raw (Fig 6), SeSAME-preprocessed (Fig 6), or SeSAMe + ComBat adjusted beta values (Fig 6). Interestingly, in contrast to the consistent scalar biases observed in FIs, chamber number appears to impact beta values with a much larger magnitude in probes with low intensity in the green channel, or high intensity in the red channel. Together, these results suggest that positional biases may strongly impact methylation beta values, most particularly in probes where the red channel FI is high and/or the green channel FI is low.

### Relationship between variability in low-level data and epigenetic clock predictions

As we had observed intensity-dependent biases with regard to both fluorescence intensity and beta values, we hypothesized that these biases in fluorescence intensity and other low-level data might underlie the variability in beta values. To explore this, we calculated the standard deviation of SeSAMe-preprocessed beta values across all replicate samples for each subject in the study, and then measured the correlation between each of several low-level data features and the standard deviation of beta values (S6 Fig). We observed that the number of beads observed for each CpG was inversely correlated with the variability in observed beta values. That is, as the number of beads measuring a CpG increased, the standard deviation of the beta values decreased. Additionally, we observed that the intensity in the green and red channels were also related to variability in beta values, suggesting that data quality might be improved by adjustments for intensity bias.

These results suggest that batch effects and other biases play a substantial role on the overall variability of methylation data, even after common preprocessing methods. However, it remained unclear how or whether such variability is meaningful for common predictive modeling applications using DNA methylation data. To examine this further, we asked whether the variability in predicted ages generated by epigenetic clocks might be explained by variability in low-level data.

**Fig 7:**
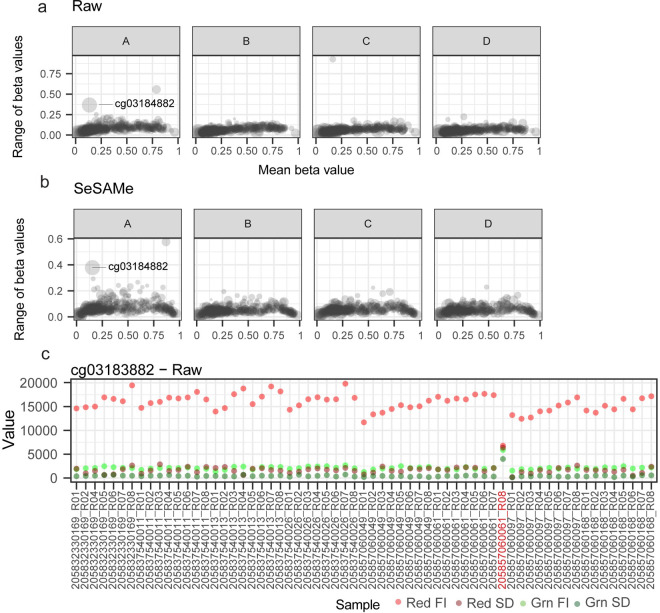
Variability in FI for epigenetic clock CpGs. (A-B) Scatter plots displaying the mean vs range of beta values for CpGs included in the Horvath Skin and Blood clock. Each point represents summary statistics for a single CpG, calculated across technical replicates from one of the four subjects in the study. The size of each point is proportional to the coefficient value in the clock model. (C) Summary statistics for low-level measurements of cg03184882 within each sample in the study. The outlier sample is highlighted in red.

**Fig 8:**
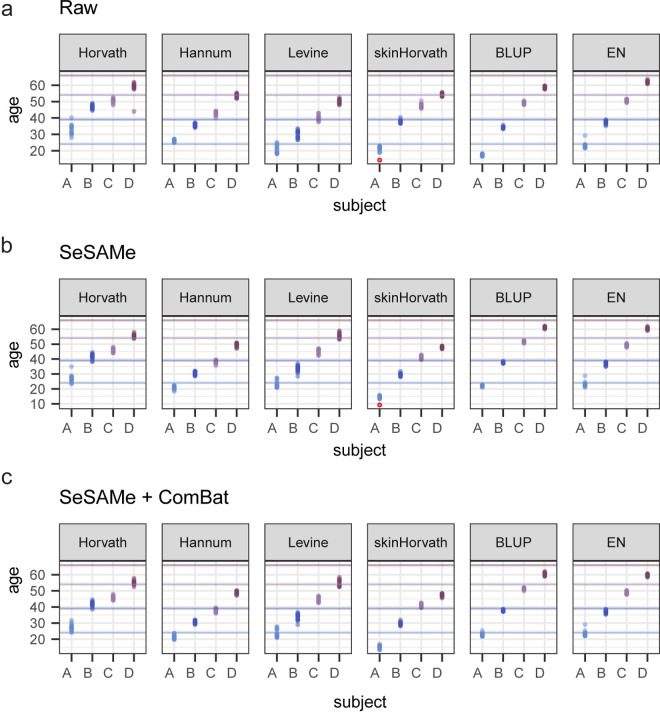
Variability in DNA methylation clock age predictions from technical replicate samples. Scatter plots depict age predictions from 6 different DNAm clocks on technical replicates. Colors of dots and lines indicate predicted age and actual age of subjects, respectively. Red point indicates outlier sample highlighted in panel C. (A) Age predictions generated using raw beta values. (B) Age predictions generated using beta values processed with the ‘recommended’ SeSaMe preprocessing settings. (C) Age predictions generated using SeSaMe-preprocessed beta values, with chamber number batch effect correction applied using ComBat.

To accomplish this aim, we first aggregated predictions from several epigenetic clocks reported in the literature, including Horvath’s clock [22], Hannum’s clock [23], Horvath’s Skin and Blood clock [24], Levine’s PhenoAge clock [25], and two clocks from Zhang, one trained using best linear unbiased prediction (BLUP) and the other trained using elastic net (EN) [26]. As anticipated, clocks were variable in both the accuracy and precision of predictions (Fig 7). When examining the predicted ages from the Horvath Skin and Blood clock, we noted that age predictions from one technical replicate from subject A stood out as particularly erroneous compared to other technical replicates (Fig 7). To ascertain what might alter the predicted age from this technical replicate, we examined the variability and coefficients of CpGs within the Horvath Skin and Blood clock. Two CpGs in particular showed a high variability within subject A, in both Raw and SeSAMe-adjusted beta values (Fig 7). We chose to examine one of these, cg03183882, in greater detail due to a larger coefficient within the Horvath Skin and Blood clock (Fig 7). Interestingly, outlier testing indicates that the abnormal technical replicate was a statistical outlier with regards to fluorescence intensity in both the green (Grubbs test for one outlier; p-value < 2.2e-16) and red (Grubbs test for one outlier; p-value = 6.448e-05) channels as well as the standard deviation of fluorescence intensity in green (Grubbs test for one outlier; p-value < 2.2e-16) and red (Grubbs test for one outlier; p-value = 3.269e-10) channels (Fig 7). Interestingly, adjusting the data with ComBat appears to partially compensate for the impact of this CpG on the age predictions of the Horvath Skin and Blood clock (Fig 8). This analysis suggests that CpG outliers detectable within the low-level microarray data may drive variability seen in epigenetic clocks, and that more stringent quality control measures which consider fluorescence measurements might improve microarray performance, as well as the performance of downstream analyses.

## Discussion

In this study, we explore variability in the Illumina MethylationEPIC microarray data using multiple highly similar technical replicates, allowing us to isolate and quantify the technical variability arising from positional effects within the MethylationEPIC v1 platform in a manner that, to our knowledge, is unique within the published literature. We assume that all variance observed between pooled samples arises from technical noise or biases within the microarrays themselves, rather than any biological variability. Similarly, because of the aggregate nature of measurements using Illumina BeadChip technology, with FI measurements being derived from many probes per bead and averaged across several beads per CpG, we discount the possibility that sampling bias explains the differences between technical replicates reported in this study.

First, using principal compenents analysis, we demonstrated the occlusion of a strong biological signal by positional effects within the array. In this study, we generated technical replicates with a far higher similarity than might be reasonably expected from any experiment. Despite this, the chamber number positional effects on the samples were sufficient to result in poor hierarchical clustering of replicates from the same subject within PCA space until corrected for using ComBat. This finding has notable implications for previous studies of microarray reliability using technical duplicates.

Next, using linear mixed effect models, we quantified variability, distinguishing the overall contributions of slides and chamber numbers to the variance of methylation beta values. These inescapable positional effects are present even within small microarray experiments, and numerous methods have been developed to correct these effects. While many publications have explored these batch effects and make recommendations regarding the balancing of experimental groups across arrays and chamber numbers, the present study is the first, to our knowledge, to quantify the magnitude of these batch variables.

Our observations suggest that in raw data, the chamber number positional effect has a larger overall effect than slide on beta value variability; however, the effect of chamber number appears to be better corrected than slide effects using existing preprocessing tools such as the ComBat algorithm. This finding suggests that different balancing strategies should be utilized, depending on the analysis pipeline being used, and has immediate implications for the researcher attempting to minimize the prevalence of experimental errors due to positional effects: if the experiment is sufficiently large to allow for chamber number correction using ComBat, then prioritization should be given to balancing experimental groups across slides; and if not, then balancing groups across chamber numbers should be given priority.

Previous efforts at improving the reliability of DNA methylation microarrays have used varying approaches, including building clocks based off of principal components rather than individual CpGs. Our analyses suggest that these approaches, in the absence of appropriate preprocessing, may confuse technical biases in the data with biological signal, and may partially explain why many epigenetic clocks tranfer poorly to other datasets. Even within highly similar technical replicates and widely used preprocessing tools, we observed that positional effects from the microarray were highly represented in principal components and obfuscated biological variability. However, we note that after correcting beta values with ComBat, principal components become largely driven by biological differences between samples.

By exploring low-level data from our own and external datasets, we observed positional effects on the variability of FIs. These data suggest that at least some of the positional biases reported here persist across multiple studies and laboratories, including scanning instruments. In addition to increased variability, we also observed intensity-dependent biases in both FIs and derived methylation beta values. While partially corrected through the use of preprocessing with either SeSAMe or SeSAMe + ComBat, substantial chamber number biases remain, particularly in instances with low green-channel FI and higher red-channel FI.

We observed the presence of technical outliers within low-level data which appear to escape preprocessing tools and may impact the performance of epigenetic clocks. We anticipate that increasing the stringency of outlier filtering would likely result in increases in performance; however, the most appropriate method of detecting and filtering such outliers remains unexplored.

We also explored an observation of a technical outlier for a single CpG with a high coefficient in methylation age clock predicitons. This observation appears to be an outlier with regards to both fluorescence intensity and variability in both red and green channels, and these data were recorded from relatively few beads (nBeads = 4). ComBat appeared to improve the precision of biological age predictions, including adjusting this outlier to be more similar to other technical replicates; however, such outlier values among technical replicates suggests an unmet need for quality control methods that consider within-sample variability in low-level fluorescence measurements, perhaps utilizing bead-level data or the standard deviation measurements from bead-level data that are contained in idat files.

We note here the apparent benefits of preprocessing with SeSAMe using the authors’ recommended settings, as well as chamber number batch adjustment with ComBat, for several metrics of variability. While these processing steps dramatically reduce the number of false positive findings on differential methylation tests while preserving detectable biological variability, a large number of preprocessing tools and normalization stragies exist which attempt to correct for the biases noted here, among other issues. While it is not feasible to exhaustively examine all possible preprocessing pipelines, we hope that this publicly available data will allow future researchers insight regarding the false discovery rate and positional biases of novel tools. Likewise, while we note here that chamber number appears to impact fluorescence variability, and that this phenomenon appears to be present in data from several iScan instruments, it is possible that this is not uniform across all such instruments. We expect that additional advances in analysis techniques may be possible to address some of the shortcomings noted in this study, particularly with regards to correcting for positional biases.

A limitation of the present study is the focus on whole blood samples. In deciding which specimen type to measure for this study, we gave primary consideration to the prevalence of specimen types within existing studies. While several clocks have been created using methylation datasets drawn from mutliple tissue types or resistant to cell-type specific effects [4,27–30], whole blood remains the preeminent specimen type in both academic research and commercial applications of DNAm microarrays. Thus, we selected this tissue to maximize the utility of the findings. As we do not consider here potential interactions between technical variability and specimen type, we urge caution for those attempting to extrapolate the variability measurements calculated here to other tissues.

Additionally, it is possible that batch correction methods such as ComBat might perform ususually well in the present study given the highly replicated experimental design. Finally, we note that while preprocessing with ComBat reduces variability in the methylation data, it does not reduce variability in the epigenetic-clock predicted ages of our technical replicates, perhaps because the positional effects noted here are partially attenuated by the feature selection process.

In this manuscript, we identified positional effects at the lowest levels possible using standard idat files. Specifically, these files contain the mean fluorescence intensities across beads for each probe, and the variability of fluorescence intensities. Whether we are considering biases in beta values themselves, or the variability of beta values, the positional biases in detected fluorescence data directly explain the biases in the derived methylation beta values. However, it is possible that future examinations of bead-level data, which are not included within idat files, might be able to further elucidate the origins of positional biases in fluorescence intensity and variability we have observed here. In conclusion, while current preprocessing and batch correction methods like SeSAMe and ComBat provide substantial improvements over raw data for hypothesis testing and research applications, further optimization of preprocessing steps may hold significant potential to not only address positional biases discussed here, but also to enhance the performance of predictive models utilizing methlyation data.

## Materials and methods

### Sample collection

For this secondary study, whole blood samples from four adult males were purchased from Bio-IVT. All identifying information was removed from samples except for approximate age and ethnicity before sample delivery, and authors had no access to participants or identifying information either during or after data collection. Specimens used in this study were collected under a primary, IRB-approved protocol and informed consent was obtained under the primary protocol which specified that samples could be used for future research, including genomics research and sharing of genomics data as conducted here. No further IRB approval was obtained for this study.

Donors were screened and found to be hepatitis B surface antigen negative, HBV NAT negative, HIV 1&2 antibody negative, HIV NAT negative, HCV antibody negative, HCV NAT negative, syphilis negative, West Nile virus NAT negative, and certified to have been negative for antibodies to *T. cruzi* either on the current or at least one previous donation. CBCs were performed and 150μl of blood was aliquoted into 1.5mL tubes and frozen on dry ice for direct shipping to Diagenode.

### DNA extraction and microarray assay

DNA extraction and microarray assays were carried out by commercial laboratory services at Diagenode. Briefly, total DNA was extracted from eight 150 μl aliquots of anticoagulated blood per subject using DNeasy Blood and Tissue Kit (Qiagen, Cat No. 69504) with RNase treatment option, using RNAse cocktail from Thermofisher (AM2286). Final elution of DNA was carried out in 200μl of elution buffer, and samples were quantified using Qubit dsDNA HS Assay Kit (Thermo Fisher Scientific). DNA quality was assessed using the Fragment Analyzer and DNF-488 High Sensitivity genomic DNA Analysis Kit (Agilent). Concentrations and Fragment Analyzer profiles are included in supplemental materials (S8 File).

Each of the resulting eight DNA aliquots per subject was further split into two aliquots, 64 in total, and independently deaminated, amplified, and fragmented. Deamination was carried out using EZ-96 DNA Methylation Kit (Zymo Research) according to Illumina’s recommended deamination protocol. An aliquot of DNA from each of eight technical replicates was combined into a pooled DNA sample for each subject. A total of eight independently isolated replicates for each subject and eight aliquots of the pooled sample for each subject were measured using Illumina Infinium MethylationEPICv1 array BeadChips (850K). The above steps were carried out by the Epigenomic Services from Diagenode (Cat nr. G02090000).

### Data preprocessing

Raw data in the form of idat files were loaded and preprocessed using the SeSAMe, R package to obtain either raw fluorescence or beta values (prep = “”) or fluorescence/beta values calculated using the recommended settings (prep = “QCDPB”) using the built-in manifest [31].

To obtain ComBat-adjusted beta values, SeSAMe-adjusted values were then further preprocessed by removing CpGs with greater than 10% NA values among all samples. Missing values among remaining CpGs were median imputed. This additional step resulted in a total 614,831 CpGs which were shared across all preprocessing pipelines discussed above. Values were then adjusted using ComBat from the sva R package [32]. To obtain ComBatFI-adjusted beta values, SeSAMe-adjusted fluorescence intensities were adjusted using the ComBat-Seq function from the sva package.

A single technical replicate from the pooled samples was removed as an outlier based on quality (205832330169_R03C01, subject C). After SeSAMe preprocessing, one technical replicate had an abnormally low rate of successfully detected probes (Grubbs test for outlier, p < 0.05). This sample was removed from all subsequent analyses.

Where indicated, fluorescence values or beta values for each CpG were converted to z-scores by centering and scaling, by subtracting the within-subject mean and dividing by the within-subject standard deviation among technical replicates using the scale function in R. Z-score analyses were filtered to include only CpGs with values complete and passing quality thresholds in >90% of samples. CpGs with non-finite z-score converted values were omitted from plots.

### Statistical analyses

#### Linear mixed effects models.

Beta values from raw data, SeSAMe-preprocessed data, or SeSAMe-preprocessed data adjusted with ComBat for chamber number batch effects were cleaned by removing CpGs with greater than 10% NA values among all samples and median imputing the remaining missing values as described above. Linear mixed effects regression was conducted using the lmer function from the lme4 R package [33], using beta values as the dependent variable, slide and chamber numbers as fixed effects, and subject as a random effect. Analysis of variance (ANOVA) was conducted on models using the F-test, to assess the importance of positional effects in explaining the variation in the beta values.

#### Principal components analysis and hierarchical clustering.

Beta values from SeSAMe-preprocessed data, or SeSAMe-preprocessed data adjusted with ComBat for either chamber number or slide number as batch effects were further preprocessed by removing CpGs with greater than 10% NA values among all samples, missing values among remaining CpGs were median imputed from all samples. The first 50 principal components were calculated using the factoextra R package (v1.0.7). For hierarchical clustering, the hclust function was used with Ward’s D2 method.

#### Differential methylation analysis.

Differential methylation testing for each set of comparison groups was conducted using the dmpFinder function from the minfi R package [34]. Note that for two-comparison tests, the results are similar to Student’s t-test. Multiple comparisons corrections were conducted using the Benjamini-Hochberg method.

#### Standard deviation ratios.

Ratios of the standard deviations for within-subject and across subject comparisons were calculated by averaging the standard deviations of technical replicates for each subject and dividing this average by the standard deviation of all replicates across all subjects with the addition of a small offset of 0.0001 to account for low variability CpGs.

#### Correlation plots.

Low level data were extracted from idat files using the R packages UMtools [35] and Watermelon [36]. Correlation matrices were calculated and plotted using the R package corrplot.

#### Epigenetic clocks.

Age predictions from epigenetic clocks were calculated using the methylclock R package for the Horvath, Hannum, Horvath Skin and Blood, Levine, BLUP, and EN clocks [37].

## Supporting information

S1 Fig PCA and Hierarchical Clustering of Beta Values After ComBat using Slide as Batch Effect. (A) Hierarchical clustering dendrogram representing the clustering of subjects based on the first 50 principal components from adjusted beta values after preprocessing with SeSAMe and ComBat adjustments for slide. (B-D) PCA plots showing clustering of subjects after ComBat adjustment by slide, colored by subject (B), chamber number (C), or array (D). (E) Box plot presenting F statistics obtained from analysis of variance (ANOVA) performed on beta values preprocessed with SeSAMe + Com-Bat using slide as batch. The box represents the interquartile range (IQR), with the median indicated by a line inside the box. Whiskers extend to the mini-mum and maximum values within 1.5 times the IQR. Data points beyond 1.5 times the IQR are plotted individually.
(TIF)

S2 Fig Differential methylation analysis of alternatively preprocessed methylation beta values from technical replicates without FDR correction. (A-D) Histograms illustrate distribution of uncorrected p-values obtained from probewise differential methylation testing on raw beta values (A), beta values preprocessed using SeSaMe’s recommended settings (B), and (C-D) SeSaMe-preprocessed beta values with correction for batch effects associated with chamber number using ComBat to adjust for either chamber number (C) or slide (D).
(TIF)

S3 Fig Positional effects on FI variability in internal and external datasets. (A-B) Standard box plots representing the type 2 probe FIs from pooled samples in the red (A) and green (B) color channels. FIs for each probe were averaged across the same four subjects for each chamber number. (C-D) Standard box plots representing CV from individual samples. (E-N) CV measurements for chamber numbers as collected from six publicly available datasets using Illumina MethylationEPICv1, comprising a total of 1362 samples. GEO dataset accession numbers are as displayed. Outlier values (>1.96 IQR) were hidden from all boxplots due to the large size of the datasets.
(TIF)

S4 Fig Beta values compared in technical replicates measured in different slides. (A) Configuration of samples within slides/chambers. The average of within-subject centered and scaled beta values for each CpG/slide were extracted from all chamber numbers on each slide as depicted. (B-D) Histograms representing the distributions of within-subject centered and scaled beta values for all probes on the MethylationEPIC array. The histograms correspond to different preprocessing steps: (B) raw beta values, (C) beta values preprocessed using SeSaMe’s recommended settings, and (D) SeSa-Me-preprocessed beta values with adjustment for chamber number batch effects using ComBat. All panels were derived from eight samples except array 205832330169, from which one sample was discarded due to low quality.
(TIF)

S5 Fig Relative brightness in technical replicates is correlated with probe intensity. (A-F) Plots depicting the difference from the median in percentile bins, scaled in fluorescence intensity units. Each point shown represents one percentile bin from one of four subjects. Raw, SeSAMe-normalized, and ComBat-corrected values are shown for the green (a, c, e) and red (b, d, f) channels, respectively.
(TIF)

S6 Fig Relationship between low-level variables and beta values. Plot depicts Pearson’s R (A) and Spearman’s rho (B) calculated for low-level variables and the standard deviation (SD) of either raw or SeSAMe-preprocessed beta values of Type 2 probes. Only probes present in both raw and Sesame-preprocessed data were used to calculate correlations.
(TIF)

S7 File Standard deviation of beta values averaged across technical replicates from four human participants. Standard deviations for each probe were calculated from SeSAMe-corrected beta values of technical replicates for each subject. The mean standard deviation was then calculated across all subjects.
(CSV)

S8 File DNA extraction and QC report. (PDF)

S9 File Complete Blood Counts for samples. Sheet 1. CBC data for participants. Sheet 2. Mapping between participant IDs used for CBC, DNA extraction and QC, and sample IDs used in methylation data.
(XLSX)
