# Supplementary figures and images for: Measuring technical variability in illumina DNA methylation microarrays
Source: PLoS One. 2025 Jul 11;20(7):e0326337. doi: 10.1371/journal.pone.0326337 (PMC12250497; doi:10.1371/journal.pone.0326337)

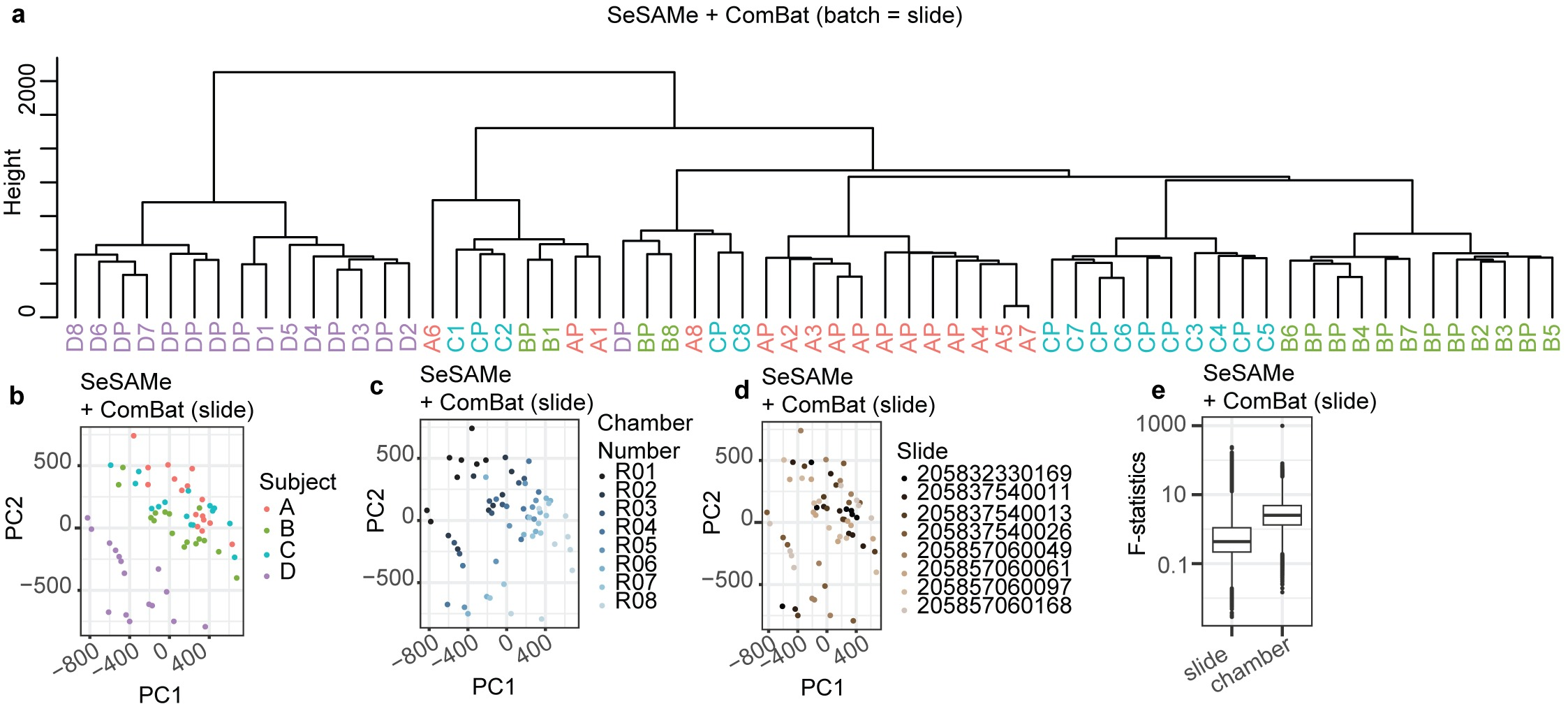

Supplement: S1 Fig — (A) Hierarchical clustering dendrogram representing the clustering of subjects based on the first 50 principal components from adjusted beta values after preprocessing with SeSAMe and ComBat adjustments for slide. (B-D) PCA plots showing clustering of subjects after ComBat adjustment by slide, colored by subject (B), chamber number (C), or array (D). (E) Box plot presenting F statistics obtained from analysis of variance (ANOVA) performed on beta values preprocessed with SeSAMe + Com-Bat using slide as batch. The box represents the interquartile range (IQR), with the median indicated by a line inside the box. Whiskers extend to the mini-mum and maximum values within 1.5 times the IQR. Data points beyond 1.5 times the IQR are plotted individually. (TIF) [file pone.0326337.s001.tif]

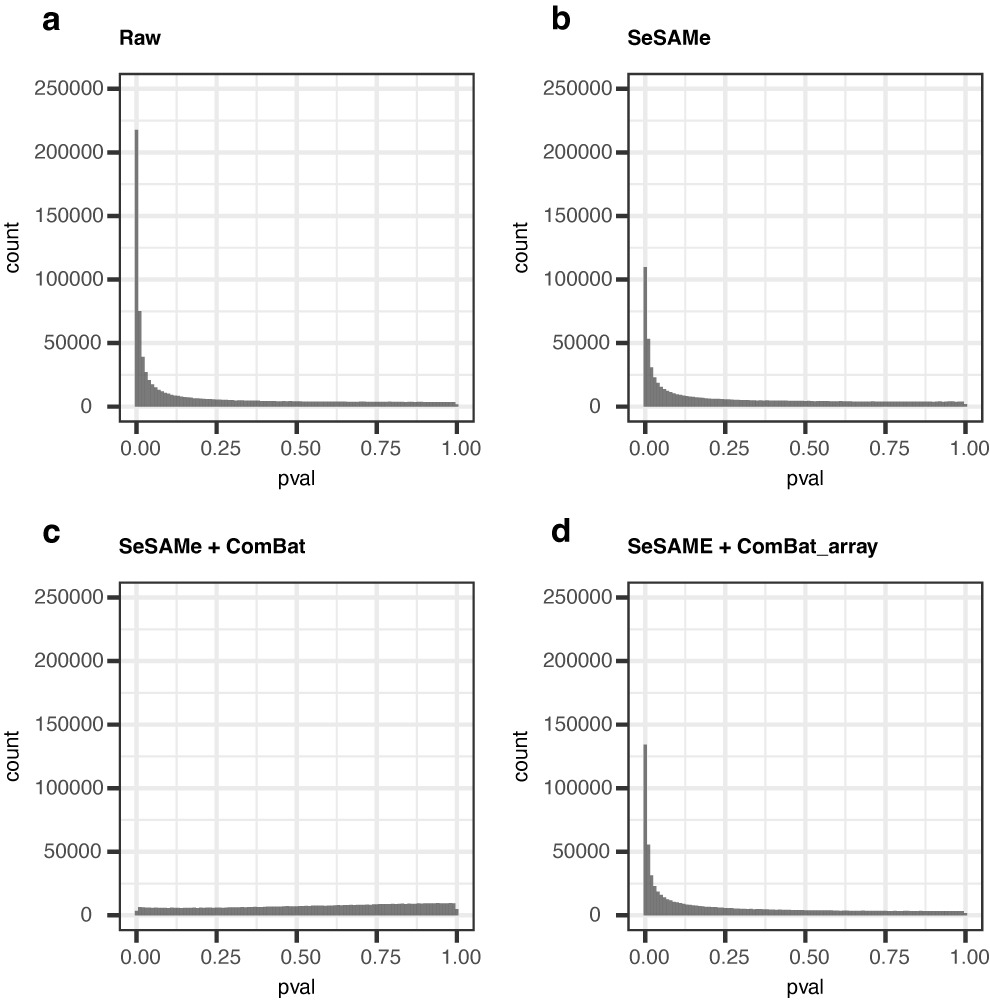

Supplement: S2 Fig — (A-D) Histograms illustrate distribution of uncorrected p-values obtained from probewise differential methylation testing on raw beta values (A), beta values preprocessed using SeSaMe’s recommended settings (B), and (C-D) SeSaMe-preprocessed beta values with correction for batch effects associated with chamber number using ComBat to adjust for either chamber number (C) or slide (D). (TIF) [file pone.0326337.s002.tif]

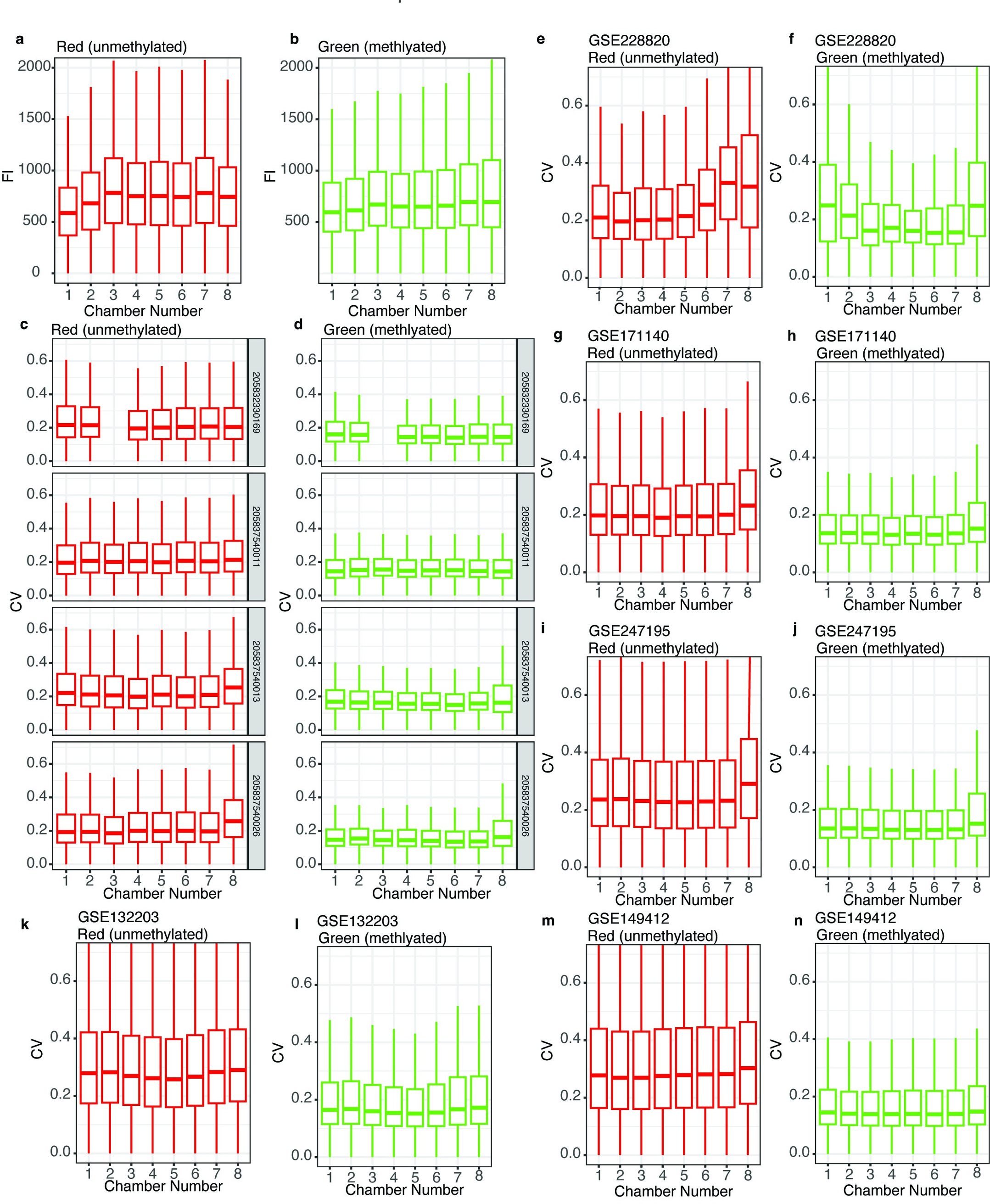

Supplement: S3 Fig — (A-B) Standard box plots representing the type 2 probe FIs from pooled samples in the red (A) and green (B) color channels. FIs for each probe were averaged across the same four subjects for each chamber number. (C-D) Standard box plots representing CV from individual samples. (E-N) CV measurements for chamber numbers as collected from six publicly available datasets using Illumina MethylationEPICv1, comprising a total of 1362 samples. GEO dataset accession numbers are as displayed. Outlier values (>1.96 IQR) were hidden from all boxplots due to the large size of the datasets. (TIF) [file pone.0326337.s003.tif]

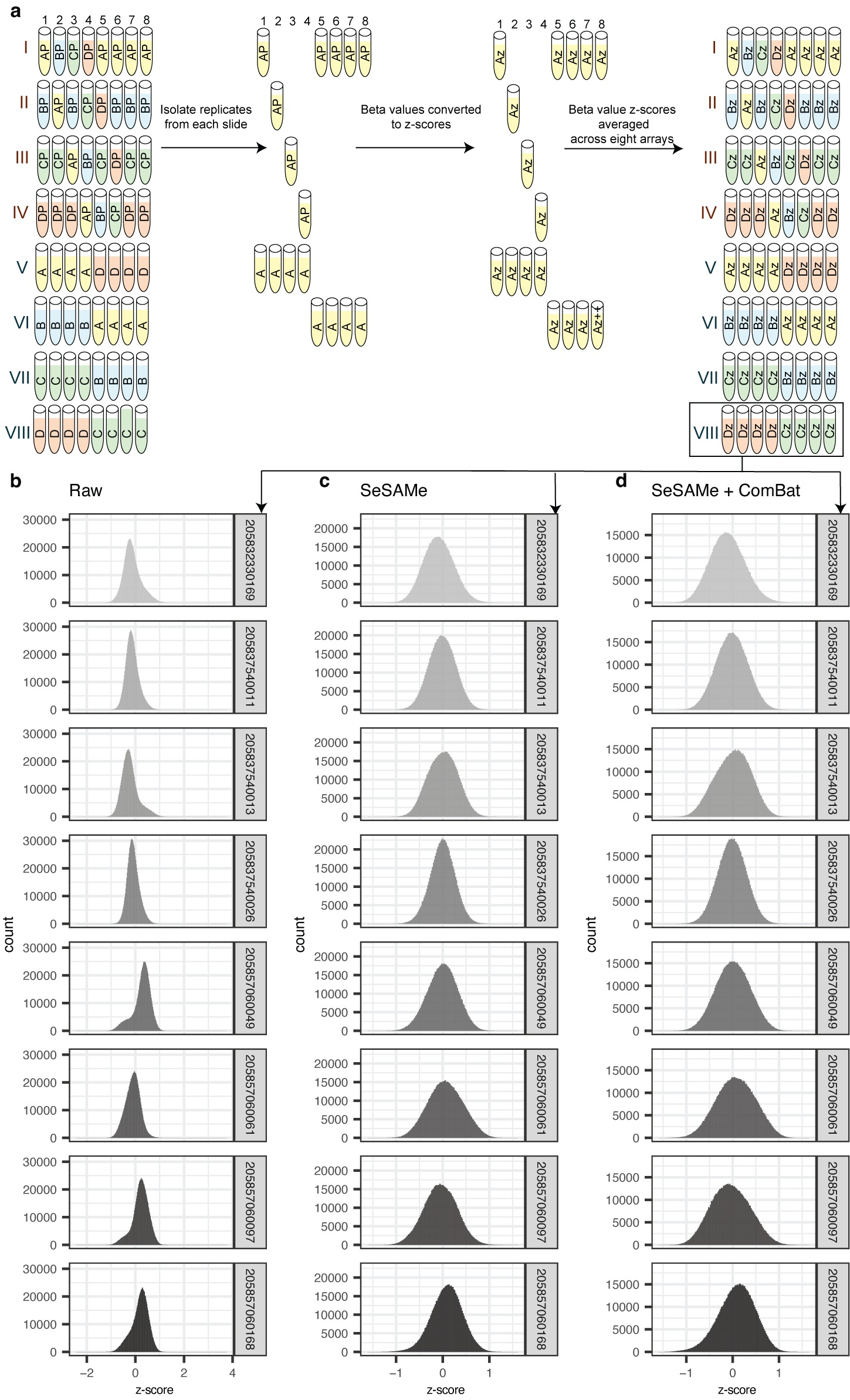

Supplement: S4 Fig — (A) Configuration of samples within slides/chambers. The average of within-subject centered and scaled beta values for each CpG/slide were extracted from all chamber numbers on each slide as depicted. (B-D) Histograms representing the distributions of within-subject centered and scaled beta values for all probes on the MethylationEPIC array. The histograms correspond to different preprocessing steps: (B) raw beta values, (C) beta values preprocessed using SeSaMe’s recommended settings, and (D) SeSa-Me-preprocessed beta values with adjustment for chamber number batch effects using ComBat. All panels were derived from eight samples except array 205832330169, from which one sample was discarded due to low quality. (TIF) [file pone.0326337.s004.tif]

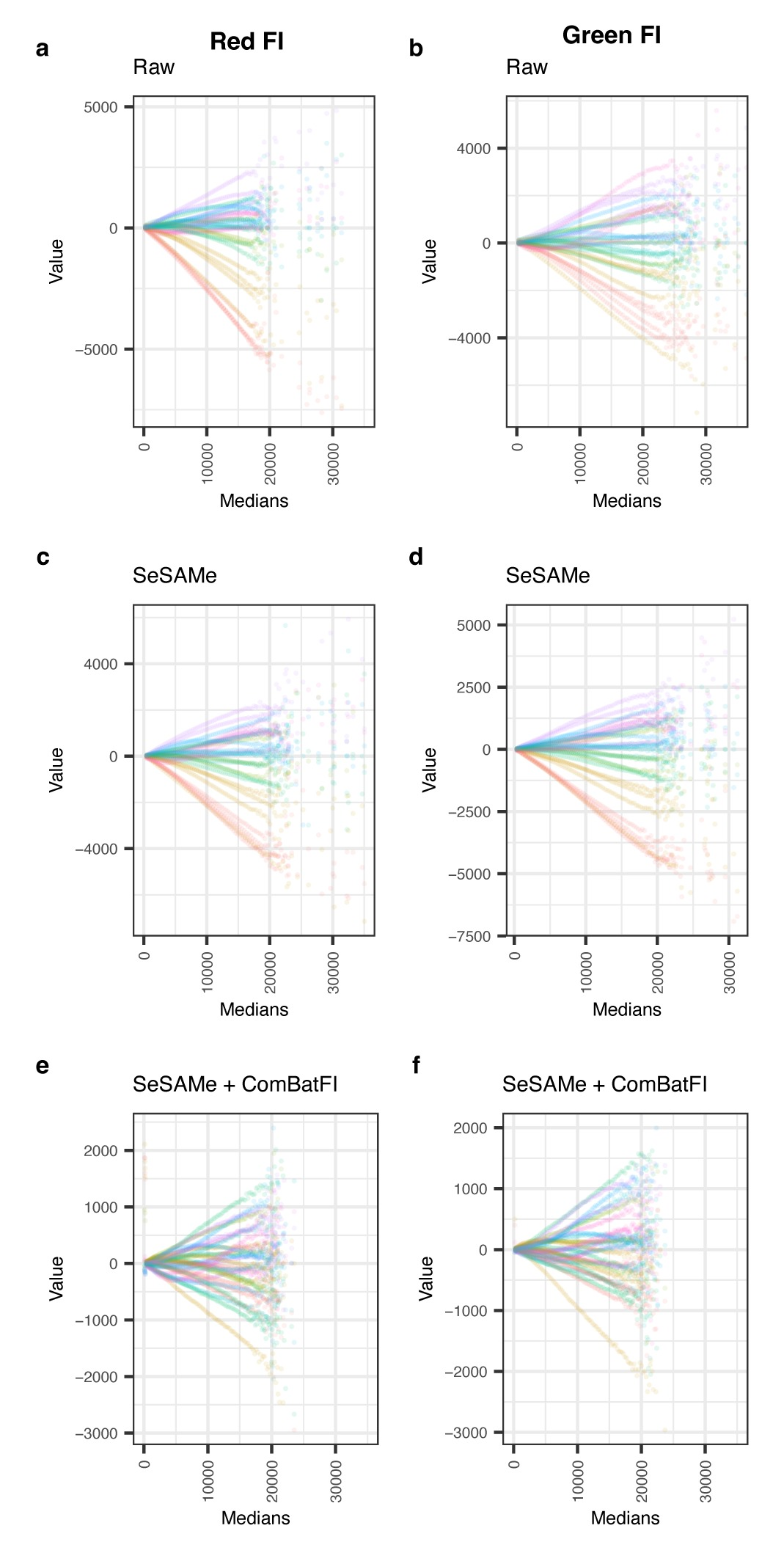

Supplement: S5 Fig — (A-F) Plots depicting the difference from the median in percentile bins, scaled in fluorescence intensity units. Each point shown represents one percentile bin from one of four subjects. Raw, SeSAMe-normalized, and ComBat-corrected values are shown for the green (a, c, e) and red (b, d, f) channels, respectively. (TIF) [file pone.0326337.s005.tif]

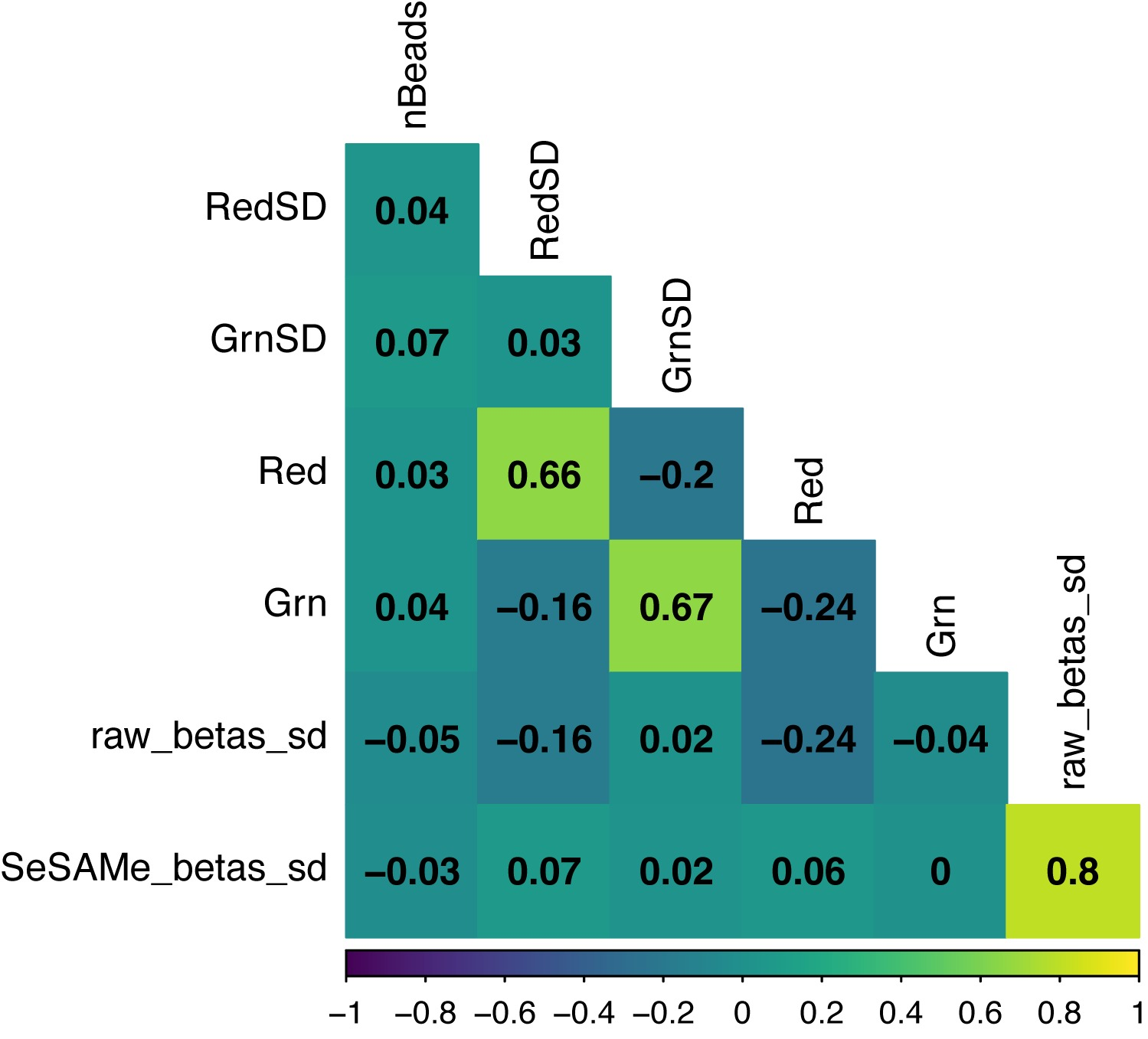

Supplement: S6 Fig — Plot depicts Pearson’s R (A) and Spearman’s rho (B) calculated for low-level variables and the standard deviation (SD) of either raw or SeSAMe-preprocessed beta values of Type 2 probes. Only probes present in both raw and Sesame-preprocessed data were used to calculate correlations. (TIF) [file pone.0326337.s006.tif]
