# Supplementary material for: Measuring technical variability in illumina DNA methylation microarrays
Source: PLoS One. 2025 Jul 11;20(7):e0326337. doi: 10.1371/journal.pone.0326337 (PMC12250497; doi:10.1371/journal.pone.0326337)
Supplement: S8 File — (PDF) [file pone.0326337.s008.pdf]

EPIC ARRAY SERVICE

Lab report

Project ID	Project Manager	Samples Received	Report sent
009_0549	KAG	18/08/2022	14/09/2022

CONTENT

1. MATERIAL AND METHODS 2
2. DNA EXTRACTION AND QC FROM WHOLE BLOOD 2
3. Terms & Conditions 14

Diagenode sa / LIEGE SCIENCE PARK / Rue Bois Saint-Jean, 3 / 4102 Seraing (Ougrée) / Belgium / Phone: (+32) 4 364 20 50 / info@diagenode.com
Diagenode Inc. / 400 Morris Avenue, Suite 101 / Denville, NJ 07834 / USA / Phone: (+1) 862 209-4680 / info.na@diagenode.com

1. MATERIAL AND METHODS

Total DNA extraction
Total DNA was extracted using DNeasy Blood and Tissue Kit (Qiagen, Cat No./ID: 69504) with RNase treatment on anticoagulated blood.

Total DNA quality control
Total DNA concentration of the samples was measured using the Qubit® dsDNA HS Assay Kit (Thermo Fisher Scientific).
DNA quality of the samples was assessed with the Fragment AnalyzerTM and DNF-488 High Sensitivity genomic DNA Analysis Kit (Agilent).

2. DNA EXTRACTION AND QC FROM WHOLE BLOOD

160 blood samples were sent to Diagenode to extract total DNA of 32 samples (4 different lots, 8 samples per lot).
Nucleid acid extraction was carried out using Qiagen DNeasy Blood and Tissue kit and a RNase treatment was performed on blood using RNAse cocktail from Thermofisher (AM2286). Final elution of DNA portion was carried out in 200µl of elution buffer.

The 32 samples were then quantified using the Qubit® dsDNA HS Assay Kit (Thermo Fisher Scientific).

Table 1 : DNA Concentration of the samples

Tube ID	Aliquots	DGN ID	Concentration (ng/ul)	Tot DNA amount (ng)
#HMN851572	A1	009_0549_001	18,3	3660
#HMN851572	A2	009_0549_002	16,4	3280
#HMN851572	A3	009_0549_003	17,1	3420
#HMN851572	A4	009_0549_004	18,4	3680
#HMN851572	A5	009_0549_005	19,5	3900
#HMN851572	A6	009_0549_006	16,7	3340
#HMN851572	A7	009_0549_007	19,9	3980
#HMN851572	A8	009_0549_008	17,2	3440
#HMN851573	B1	009_0549_009	15,9	3180
#HMN851573	B2	009_0549_010	14,9	2980
#HMN851573	B3	009_0549_011	15,4	3080
#HMN851573	B4	009_0549_012	15	3000
#HMN851573	B5	009_0549_013	15,9	3180
#HMN851573	B6	009_0549_014	15,6	3120
#HMN851573	B7	009_0549_015	15,9	3180
#HMN851573	B8	009_0549_016	16,1	3220
#HMN851574	C1	009_0549_017	14	2800
#HMN851574	C2	009_0549_018	14,2	2840
#HMN851574	C3	009_0549_019	14,6	2920
#HMN851574	C4	009_0549_020	13	2600
#HMN851574	C5	009_0549_021	13	2600
#HMN851574	C6	009_0549_022	14,6	2920
#HMN851574	C7	009_0549_023	13,7	2740
#HMN851574	C8	009_0549_024	15,3	3060
#HMN851575	D1	009_0549_025	13,2	2640

#HMN851575	D2	009_0549_026	14,4	2880
#HMN851575	D3	009_0549_027	12,7	2540
#HMN851575	D4	009_0549_028	13,8	2760
#HMN851575	D5	009_0549_029	28,8	5760
#HMN851575	D6	009_0549_030	12,9	2580
#HMN851575	D7	009_0549_031	12,8	2560
#HMN851575	D8	009_0549_032	12,8	2560

The DNA quality was then analyzed using the Fragment Analyzer™ and the DNF-488 High Sensitivity genomic DNA Analysis Kit (Agilent). The profiles are shown here below:

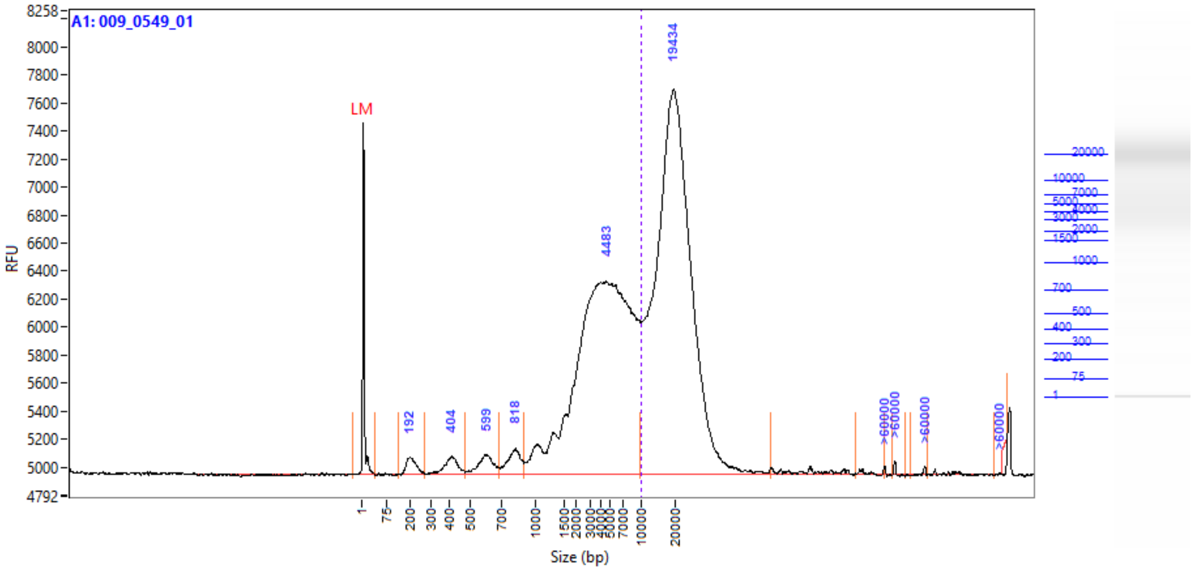

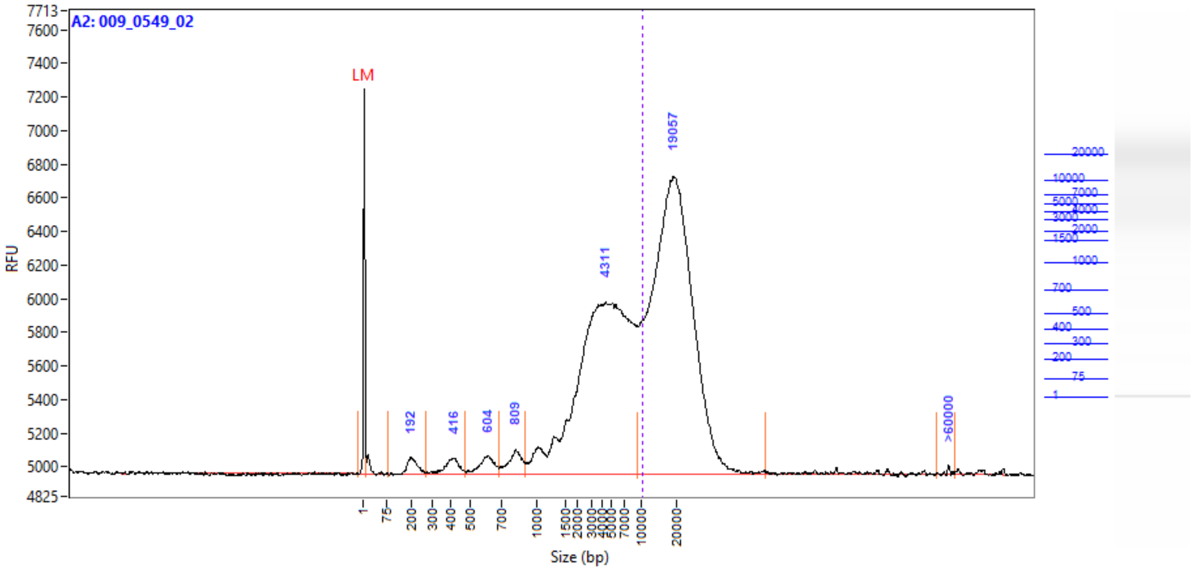

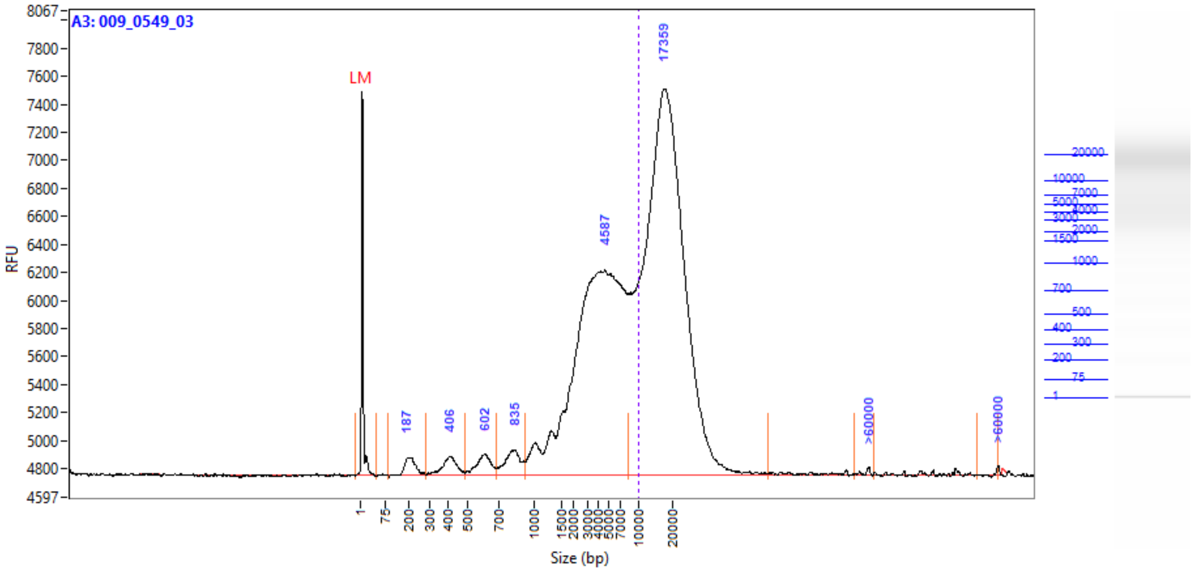
A3: 009_0549_03
RFU
LM
17359
4587
187
406
602
835
>60000
>60000
Size (bp)

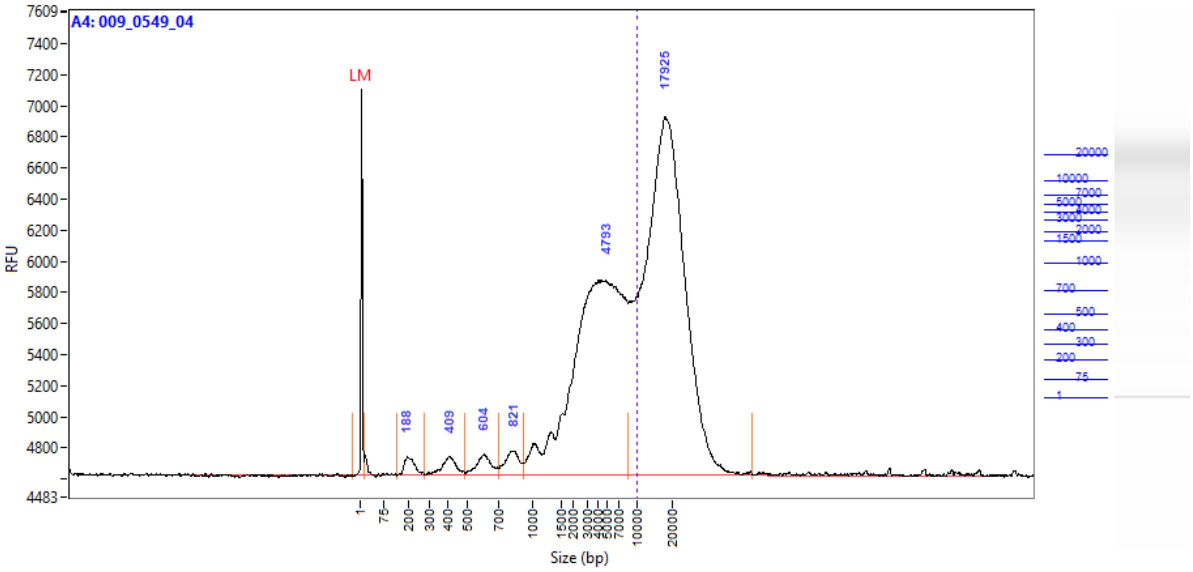
A4: 009_0549_04
RFU
LM
17925
4793
188
409
604
821
Size (bp)

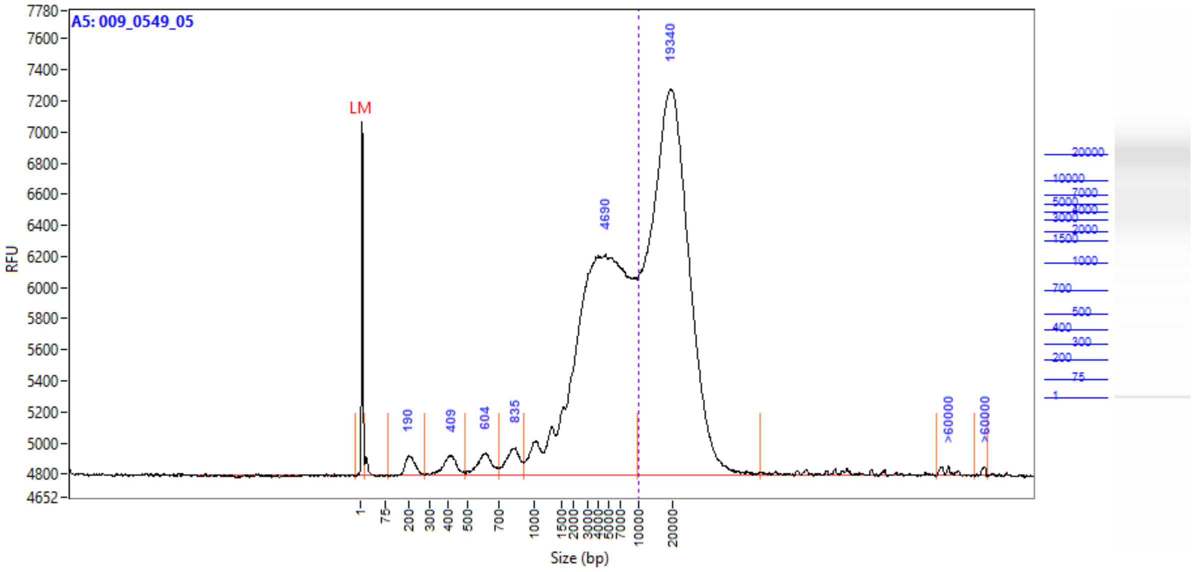
A5: 009_0549_05
RFU
LM
19340
4690
190
409
604
835
>60000
>60000
Size (bp)

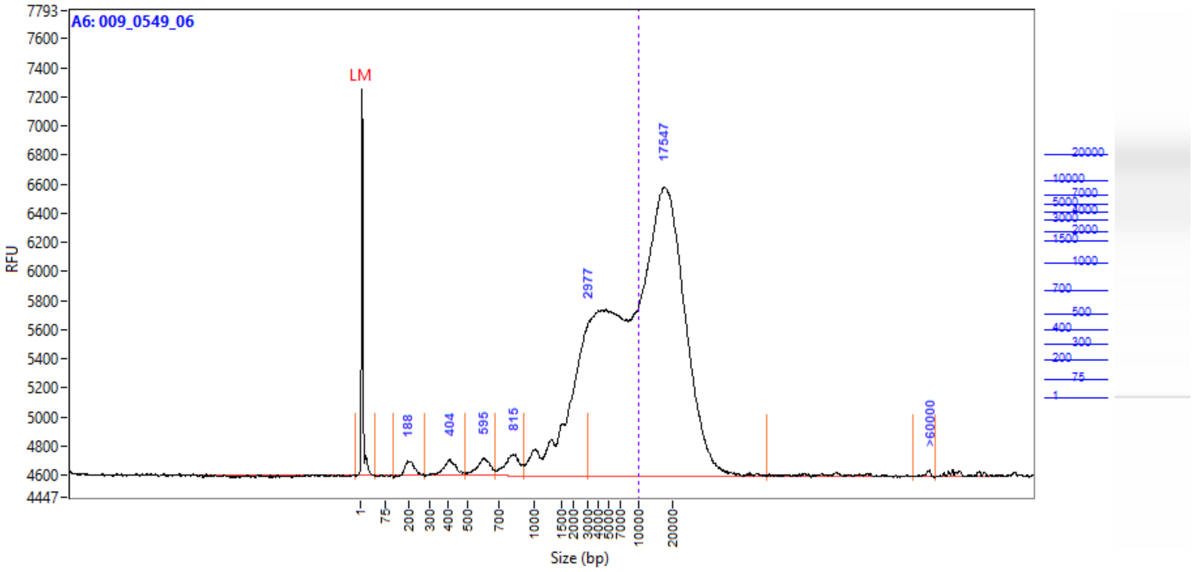
A6: 009_0549_06
LM
17547
2977
188
404
595
815
>60000
RFU
Size (bp)

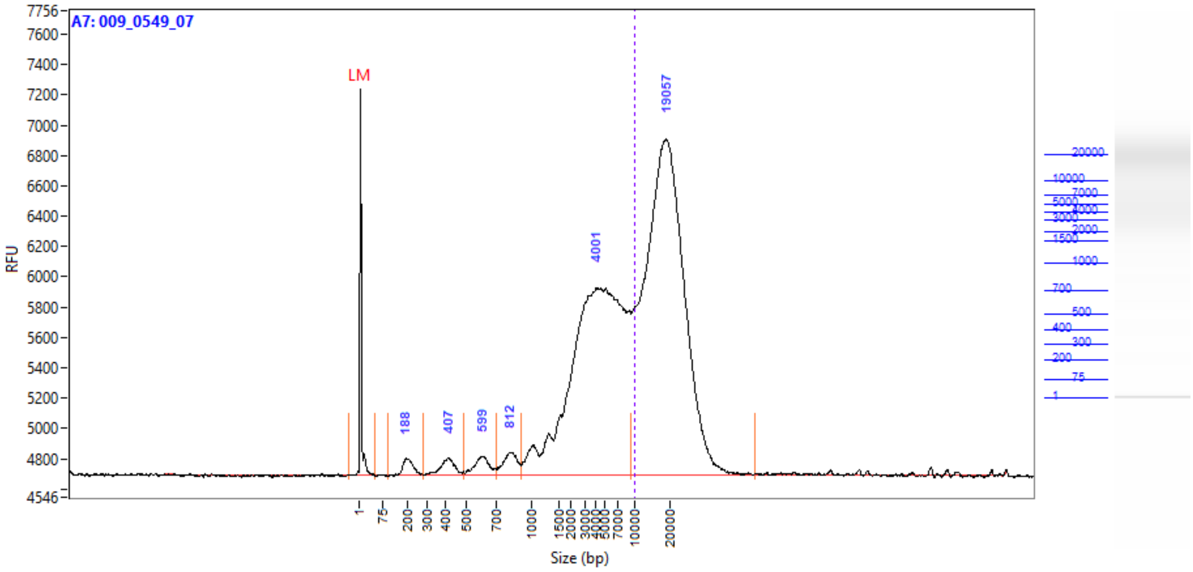
A7: 009_0549_07
LM
19057
4001
188
407
599
812
RFU
Size (bp)

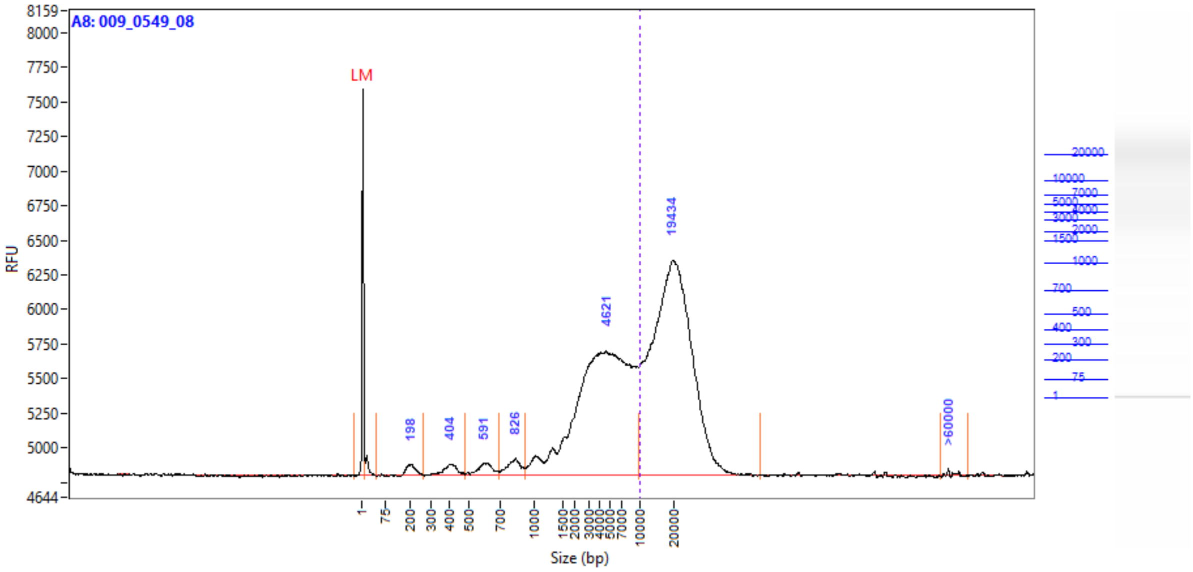
A8: 009_0549_08
LM
19434
4621
198
404
591
826
>60000
RFU
Size (bp)

diagenode
Innovating Epigenetics Solutions

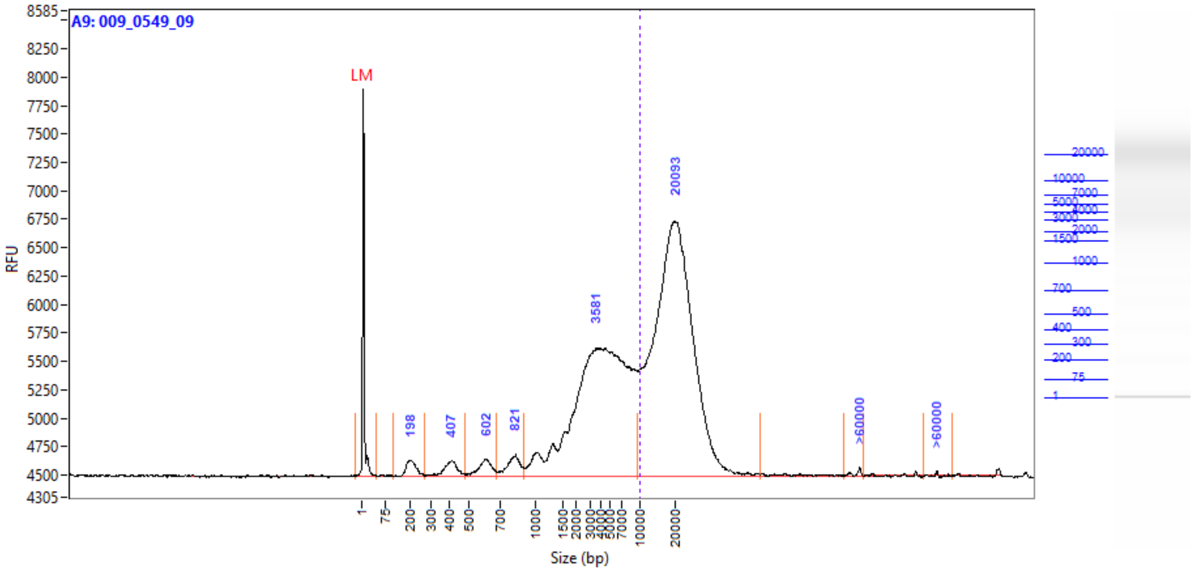

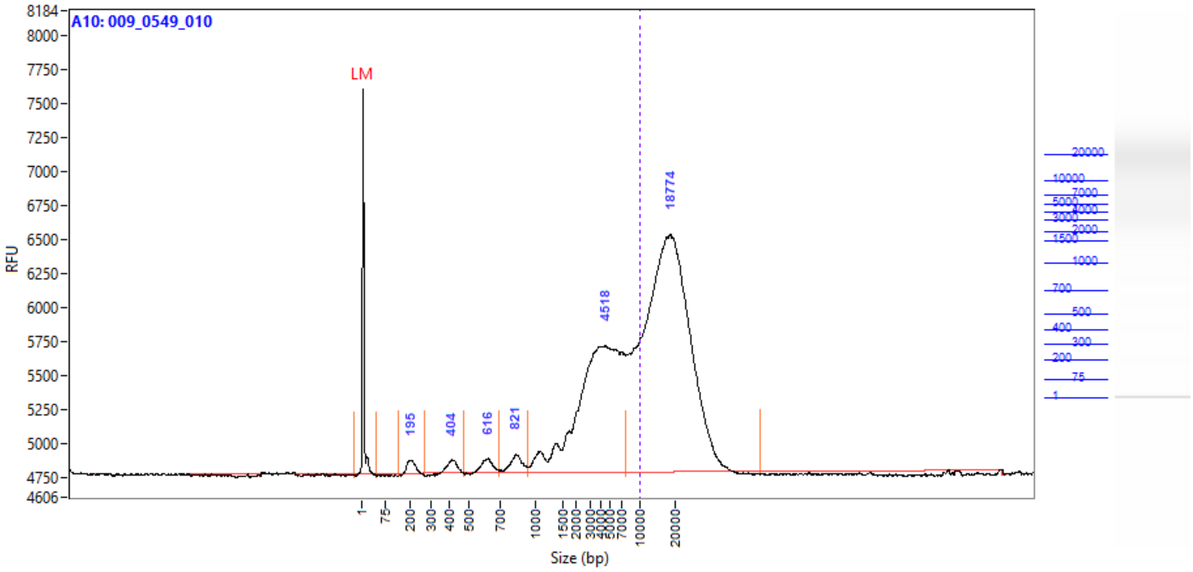

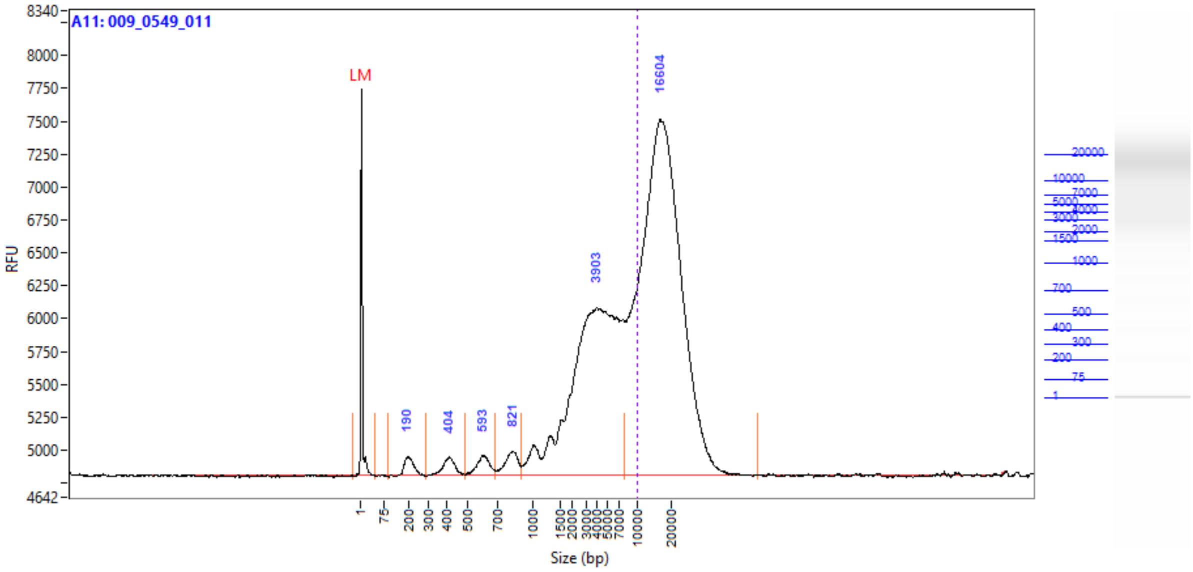

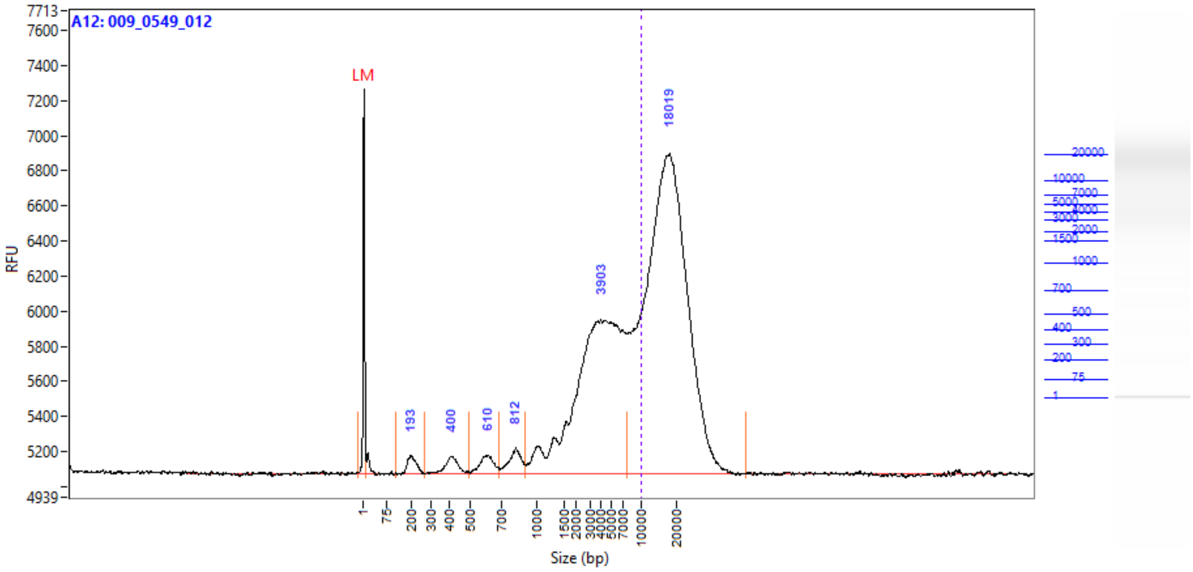
A12: 009_0549_012
LM
193
400
610
812
3903
18019
RFU
Size (bp)

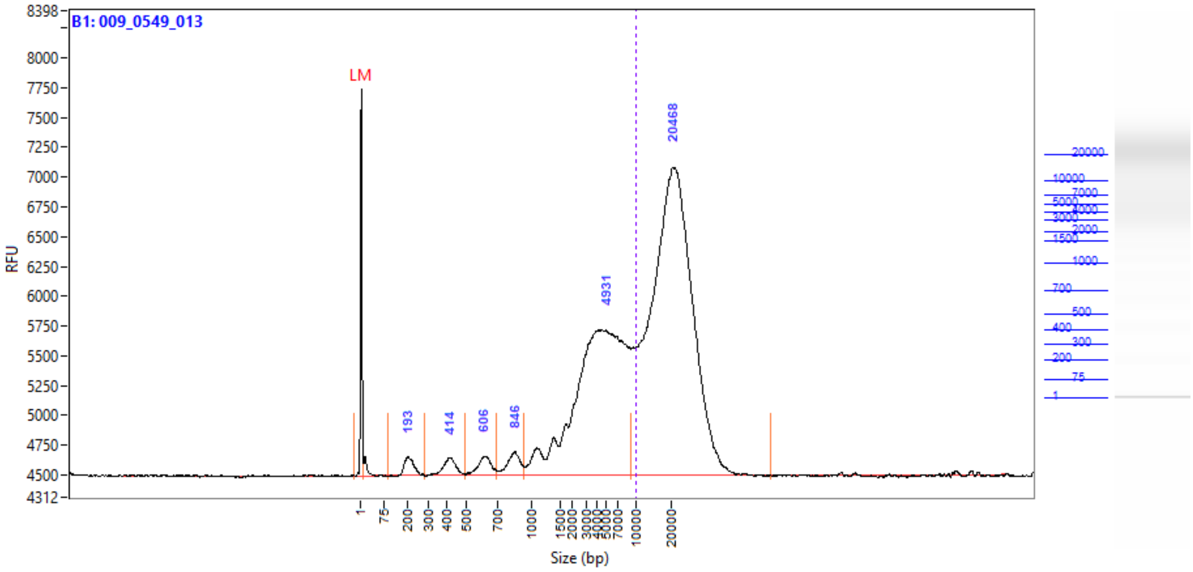
B1: 009_0549_013
LM
193
414
606
846
4931
20468
RFU
Size (bp)

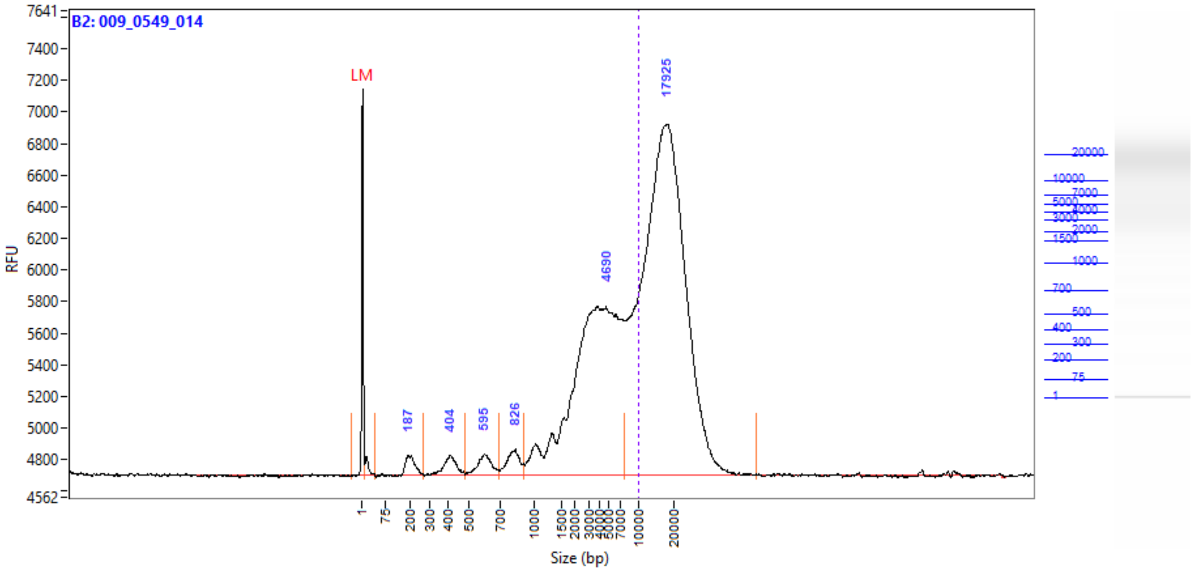
B2: 009_0549_014
LM
187
404
595
826
4690
17925
RFU
Size (bp)

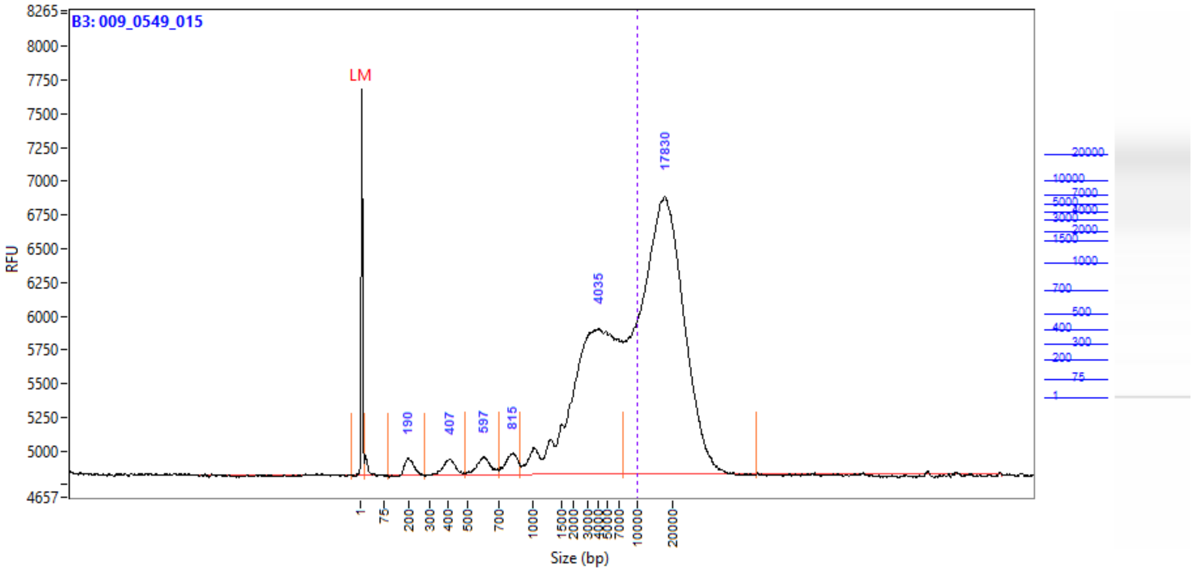

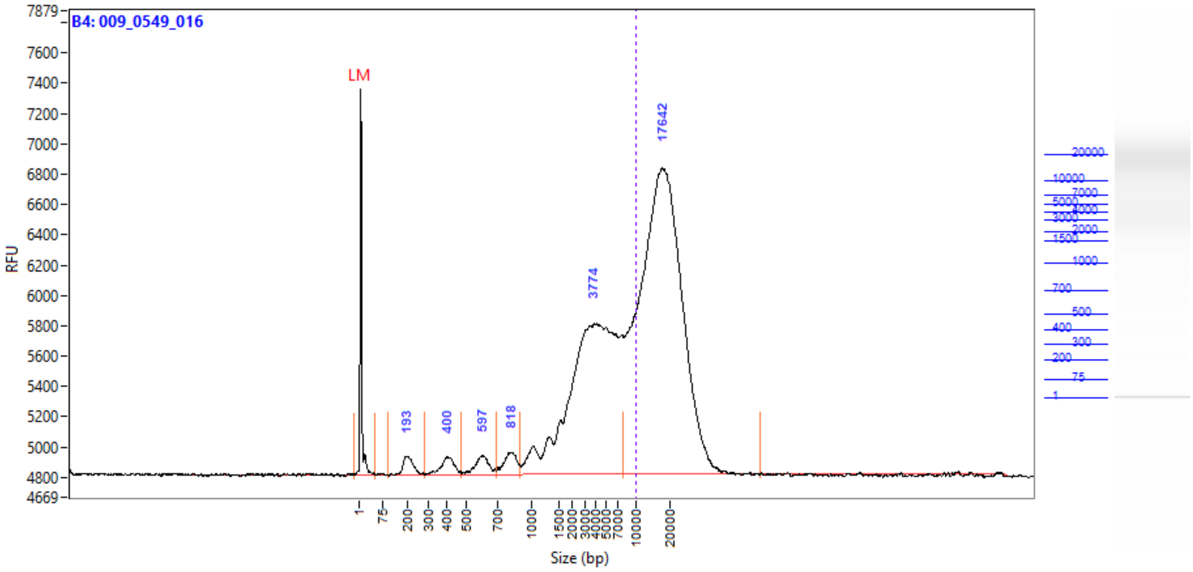

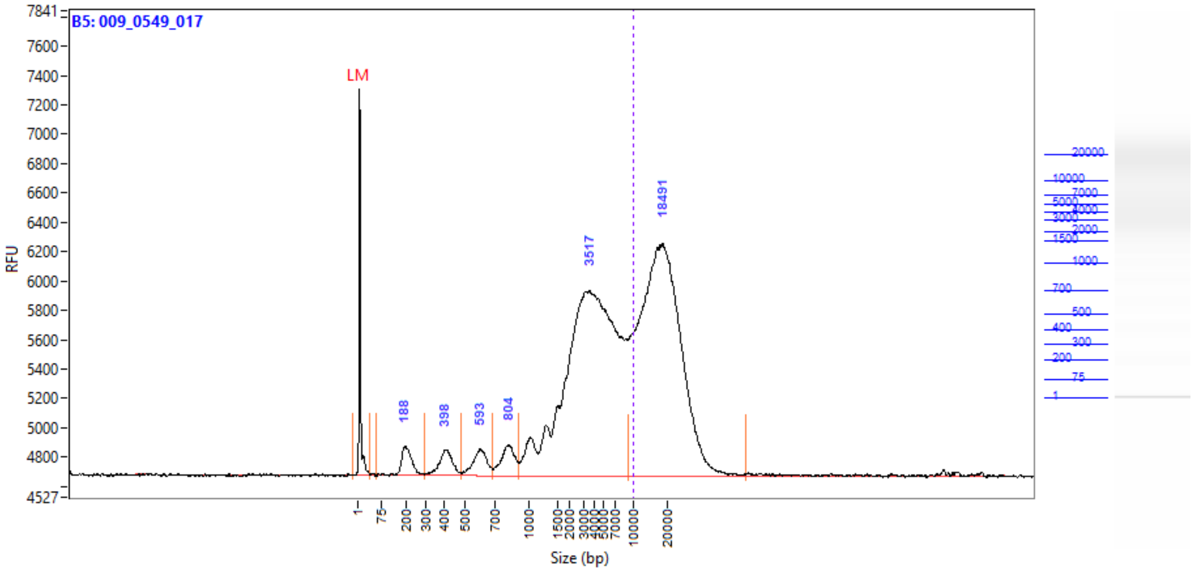

Diagenode sa / LIEGE SCIENCE PARK / Rue Bois Saint-Jean, 3 / 4102 Seraing (Ougrée) / Belgium / Phone: (+32) 4 364 20 50 / info@diagenode.com
Diagenode Inc. / 400 Morris Avenue, Suite 101 / Denville, NJ 07834 / USA / Phone: (+1) 862 209-4680 / info.na@diagenode.com

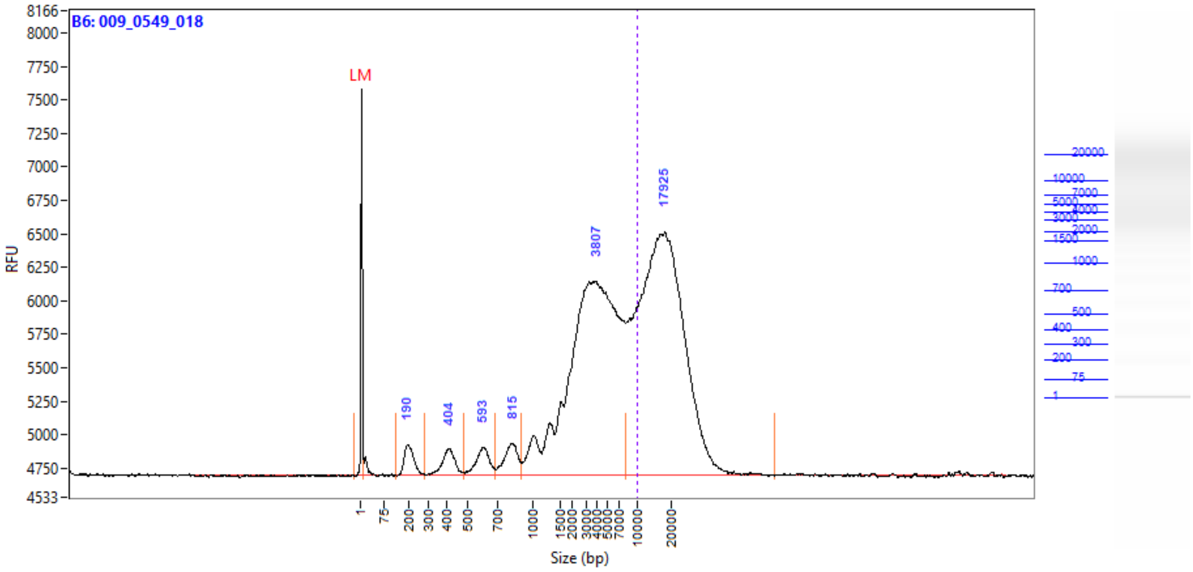
B6: 009_0549_018
LM
190
404
593
815
3807
17925
RFU
Size (bp)

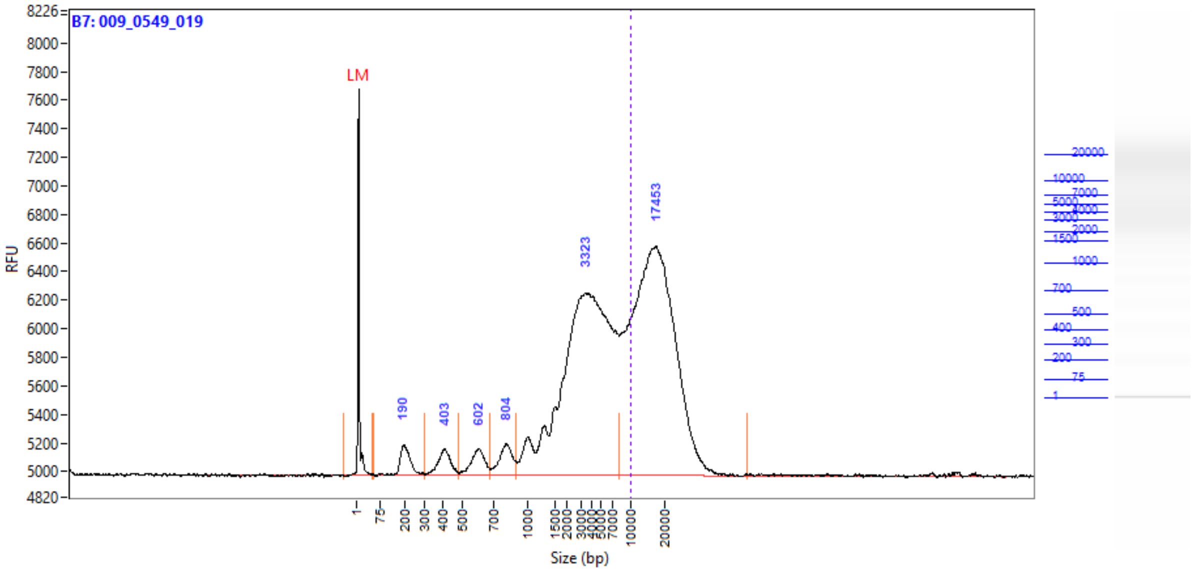
B7: 009_0549_019
LM
190
403
602
804
3323
17453
RFU
Size (bp)

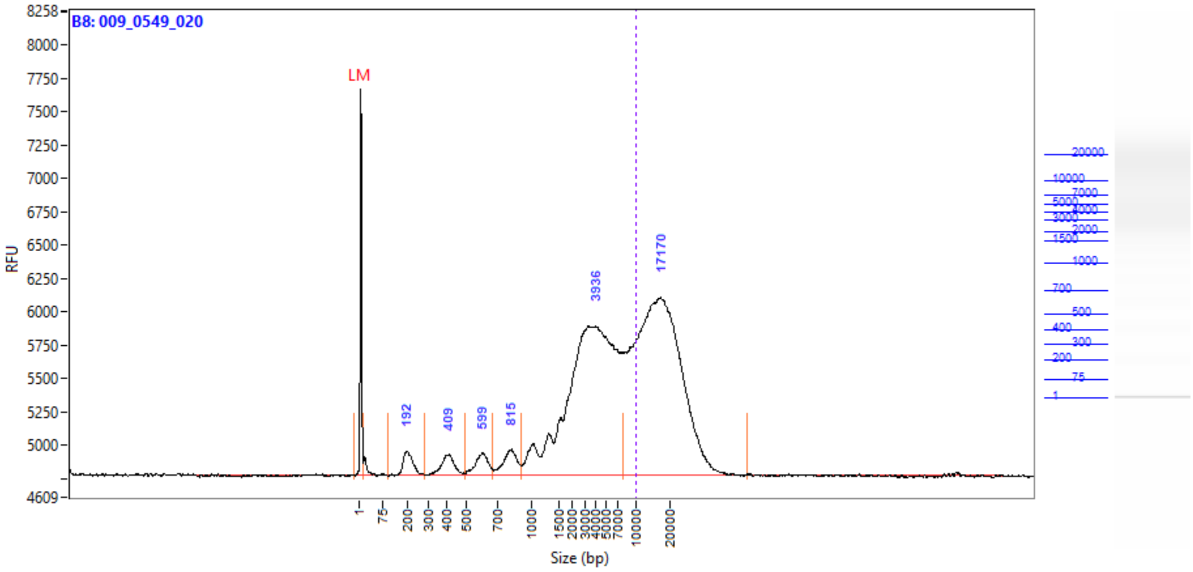
B8: 009_0549_020
LM
192
409
599
815
3936
17170
RFU
Size (bp)

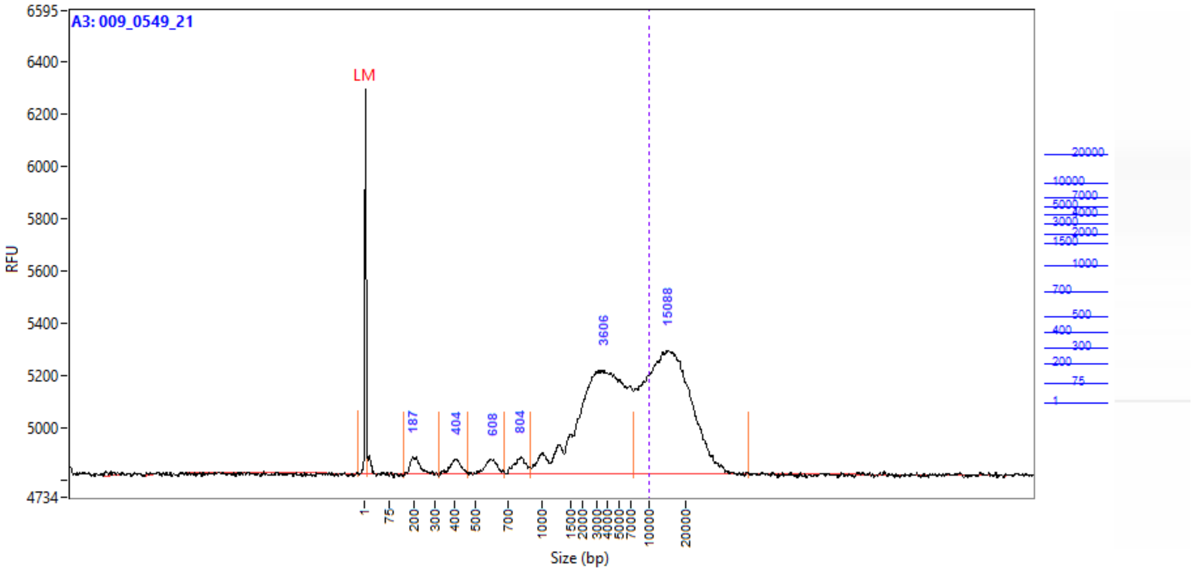
A3: 009_0549_21
LM
187
404
608
804
3606
15088
RFU
Size (bp)

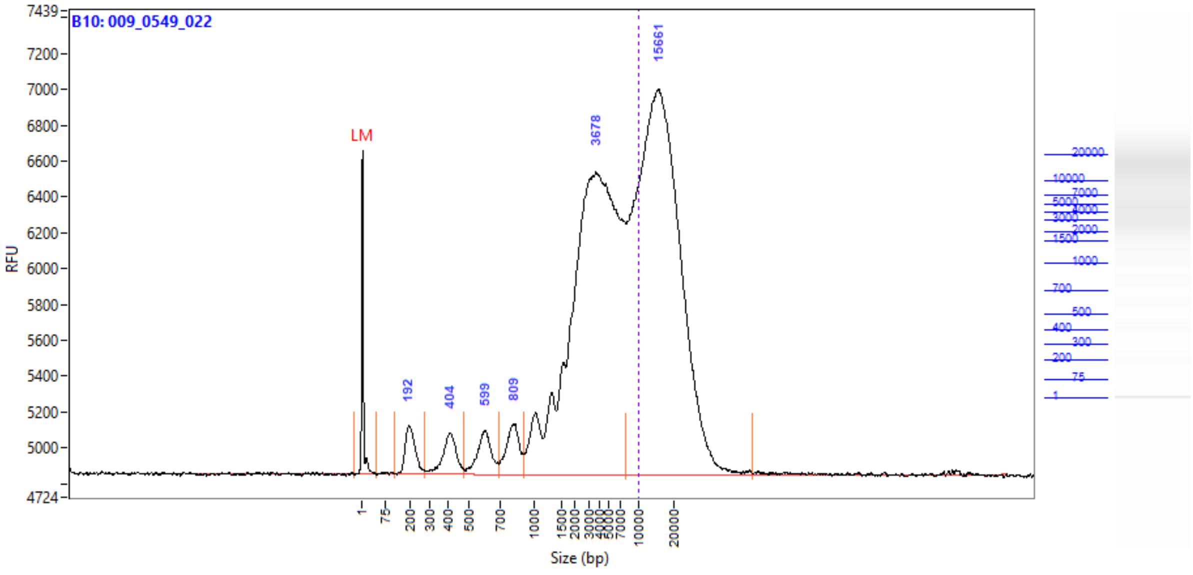
B10: 009_0549_022
LM
192
404
599
809
3678
15661
RFU
Size (bp)

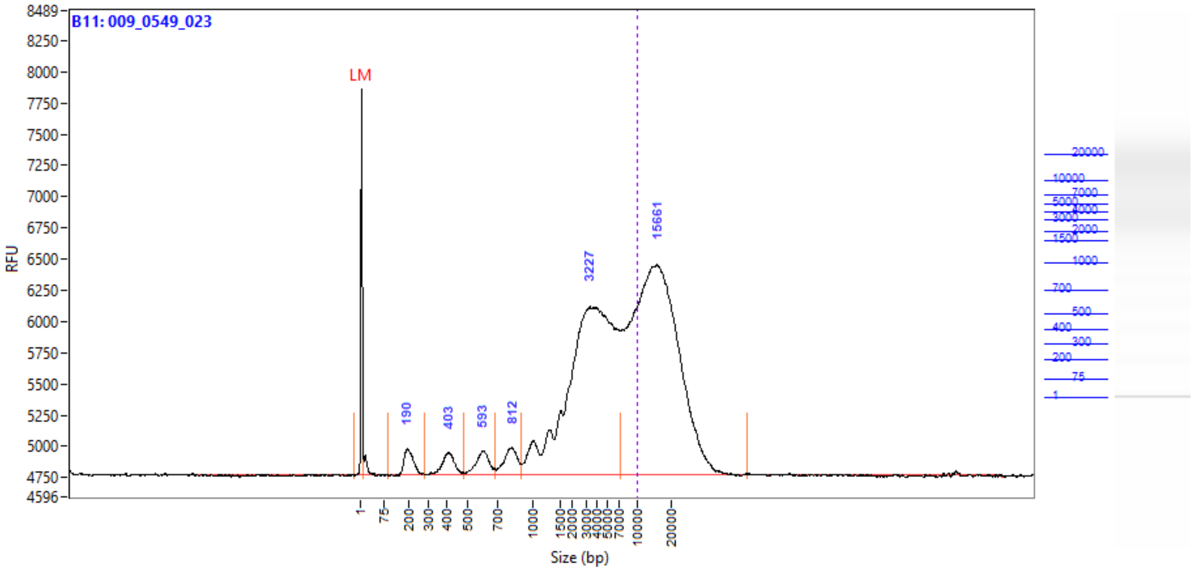
B11: 009_0549_023
LM
190
403
593
812
3227
15661
RFU
Size (bp)

diagenode
Innovating Epigenetics Solutions

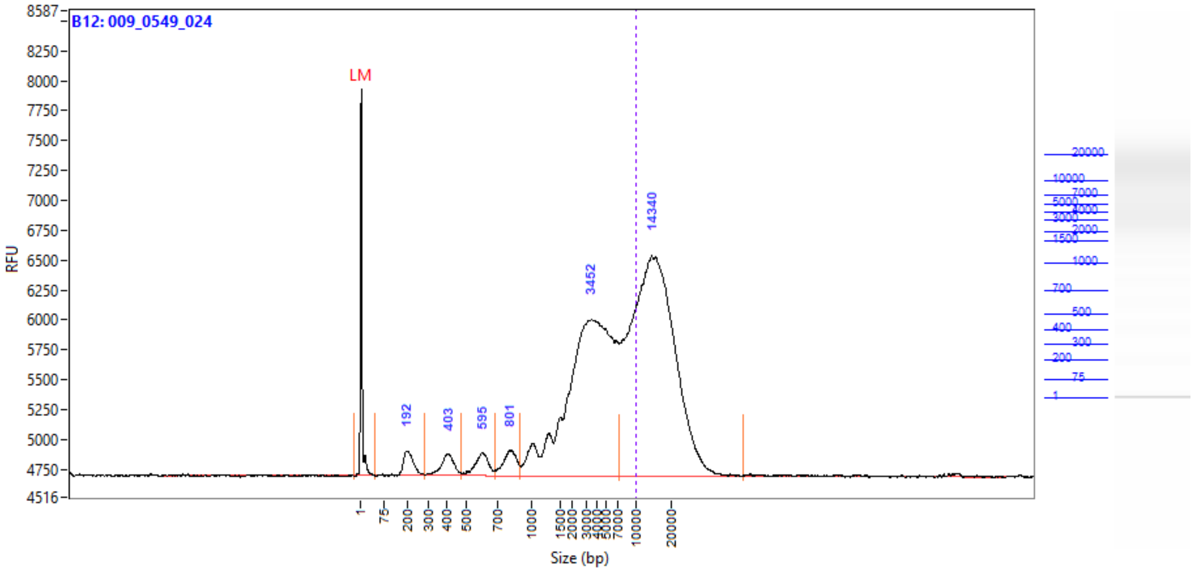

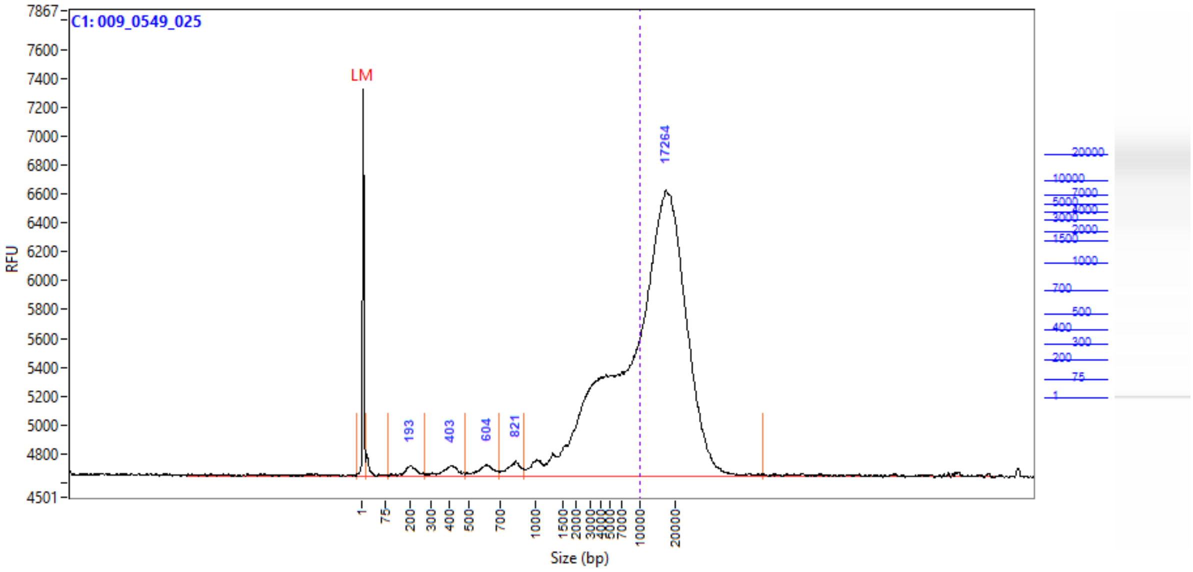

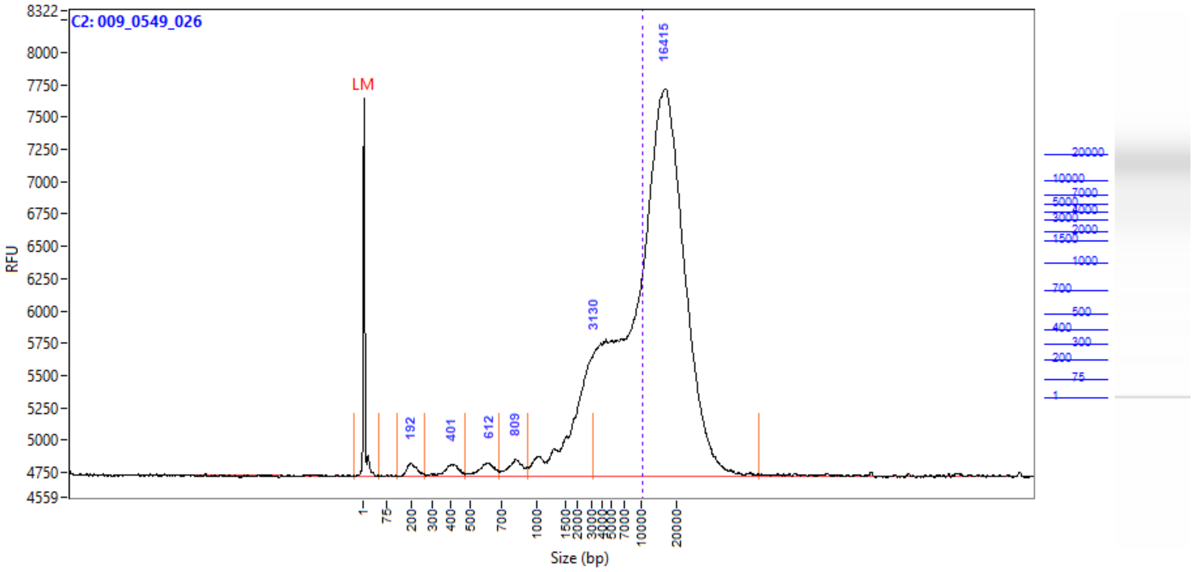

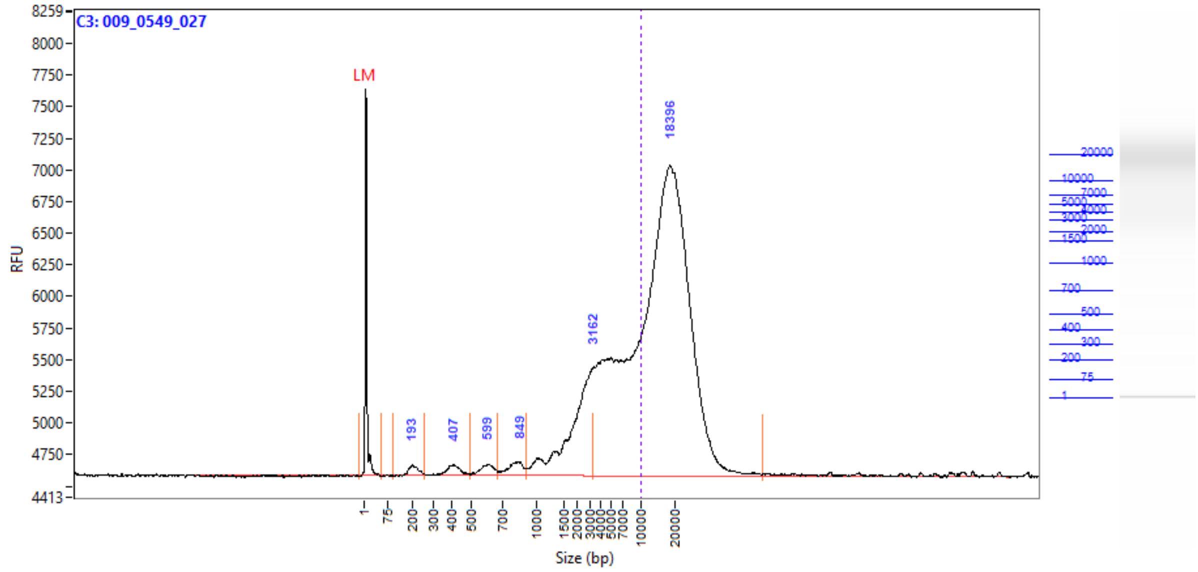
C3: 009_0549_027
LM
193
407
599
849
3162
18396
RFU
Size (bp)

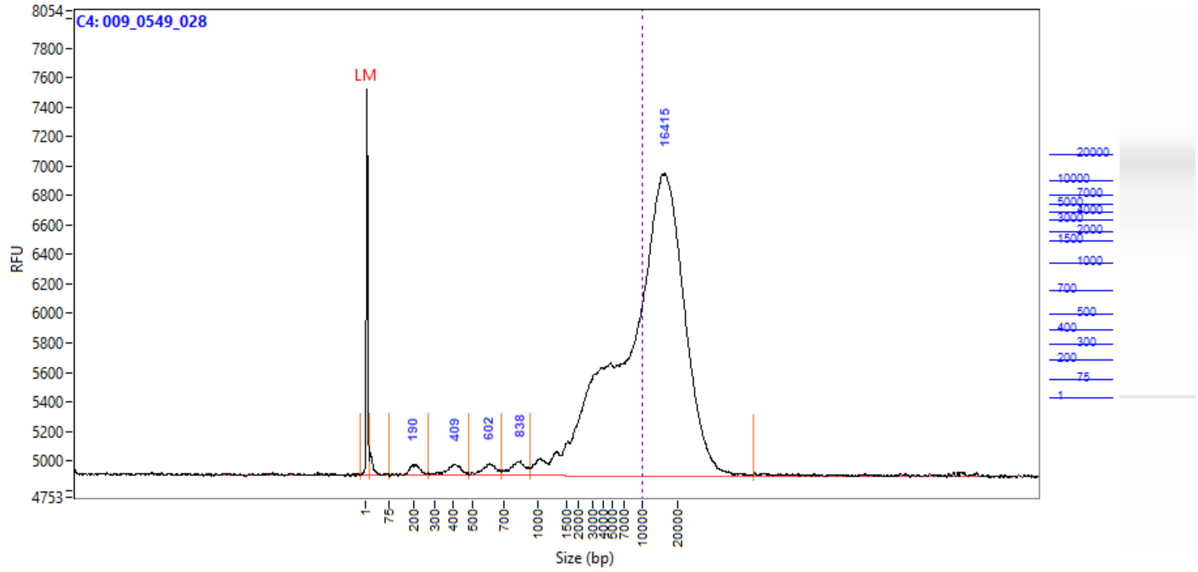
C4: 009_0549_028
LM
190
409
602
838
16415
RFU
Size (bp)

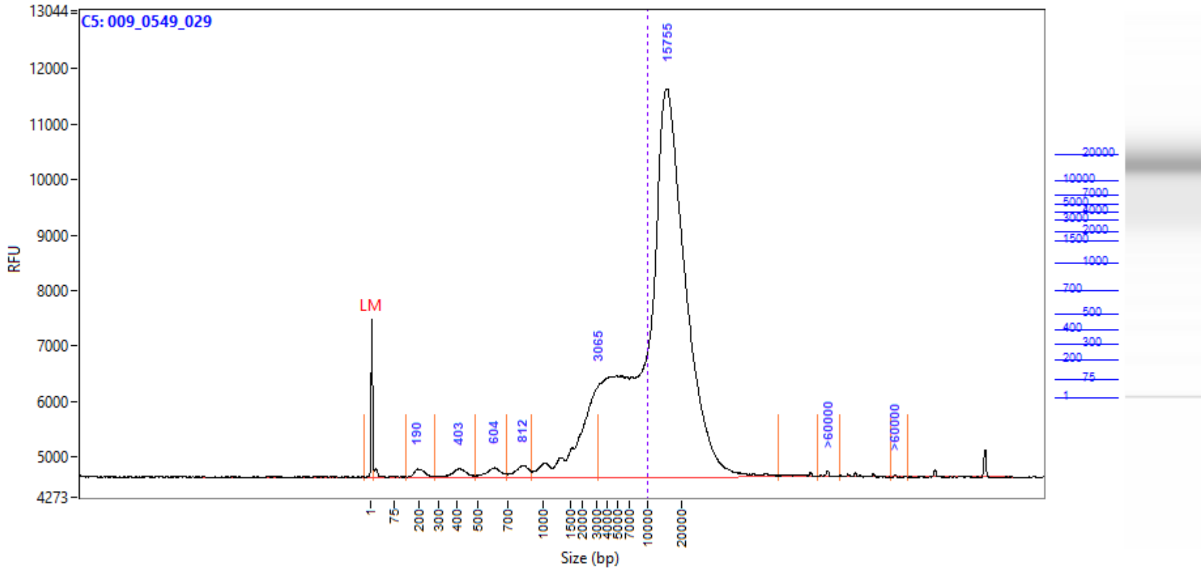
C5: 009_0549_029
LM
190
403
604
812
3065
15755
>60000
>60000
RFU
Size (bp)

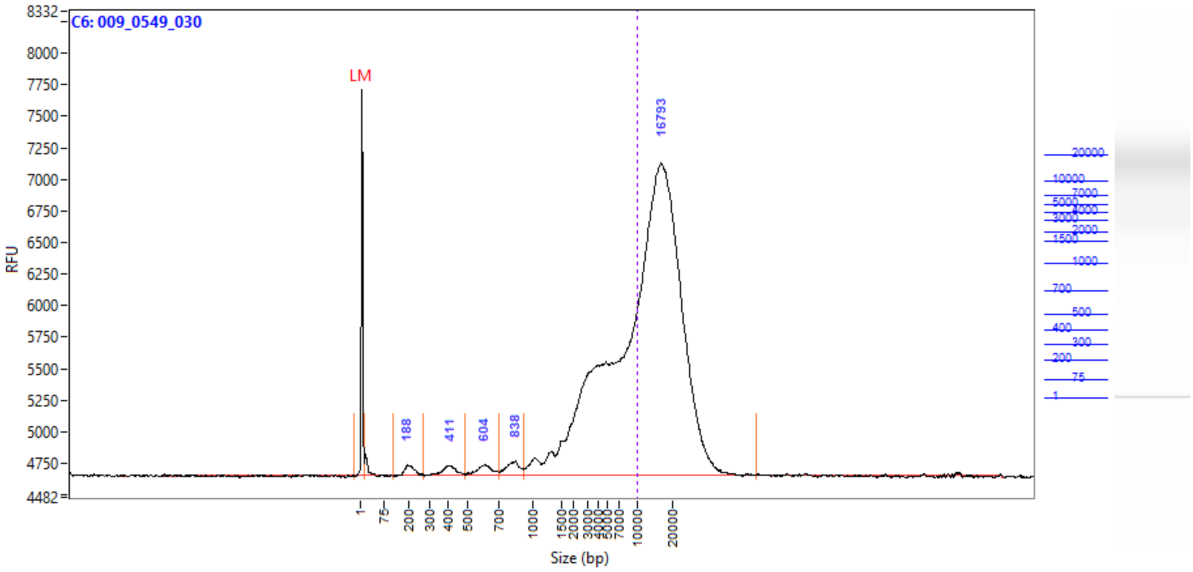

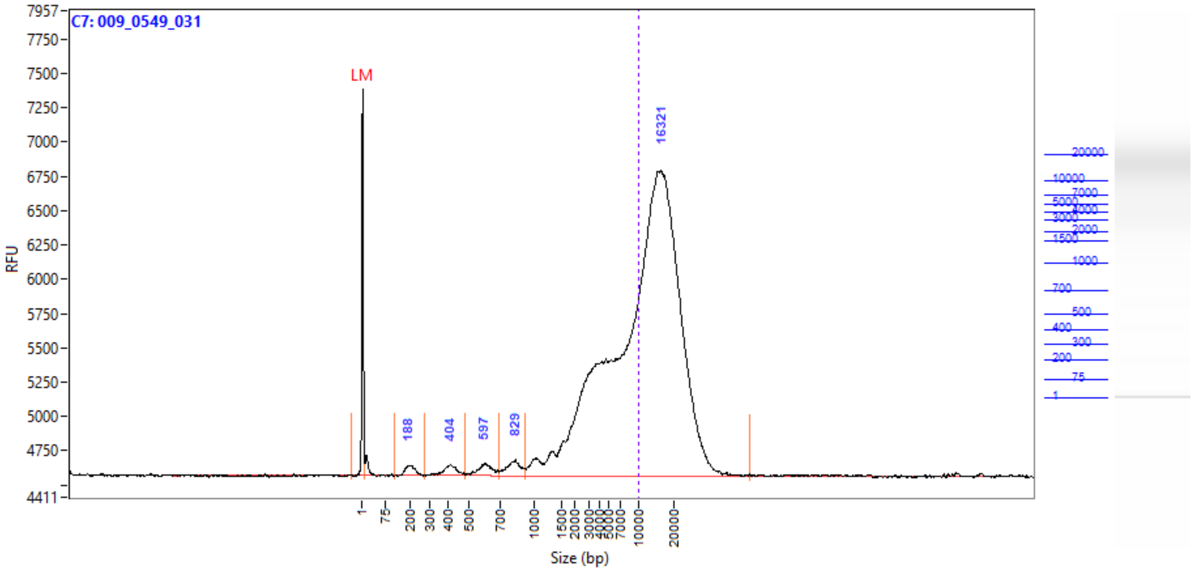

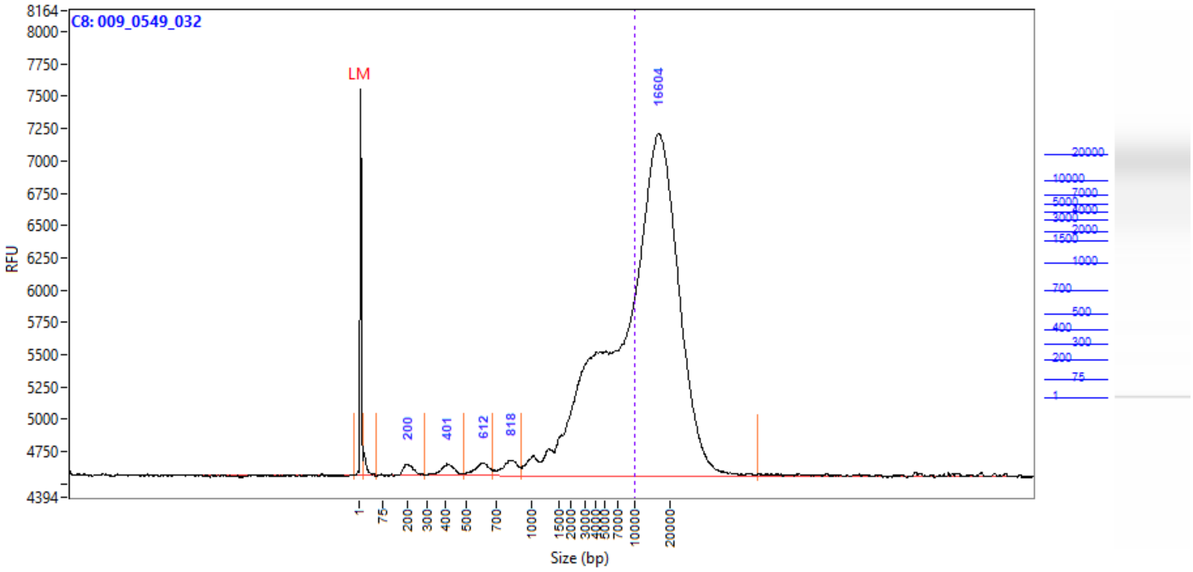

Diagenode sa / LIEGE SCIENCE PARK / Rue Bois Saint-Jean, 3 / 4102 Seraing (Ougrée) / Belgium / Phone: (+32) 4 364 20 50 / info@diagenode.com
Diagenode Inc. / 400 Morris Avenue, Suite 101 / Denville, NJ 07834 / USA / Phone: (+1) 862 209-4680 / info.na@diagenode.com

3. Terms & Conditions

Original samples are stored at Diagenode for 4 months after project completion but will be discarded once this time is exceeded. Return shipment of samples is available upon request.

Generated files will be available for download during 1 month and stored for an additional period of 3 months on Diagenode's servers. Additional long-term storage of data is available upon request.

An invoice will be issued at the completion of the service. For projects where the cost is greater than 20,000 (€, $, £), you will be billed in stages.

Diagenode works with selected third parties to arrange for data delivery and analysis services on our customer's behalf. By ordering, customer explicitly authorizes all such arrangements.
